# Evidence map of traditional Chinese exercises

**DOI:** 10.3389/fpubh.2024.1347201

**Published:** 2024-09-18

**Authors:** Yan Yu, Tongtong Wu, Murou Wu, Shaonan Liu, Xueyin Chen, Jinpeng Wu, Xinfeng Guo, Lihong Yang

**Affiliations:** ^1^The Second Clinical Medical College of Guangzhou University of Chinese Medicine, Guangzhou, China; ^2^State Key Laboratory of Dampness Syndrome of Chinese Medicine, The Second Affiliated Hospital of Guangzhou University of Chinese Medicine, Guangzhou, China; ^3^The Second Affiliated Hospital of Guangzhou University of Chinese Medicine (Guangdong Provincial Hospital of Chinese Medicine), Guangzhou, China; ^4^Institute of Biomedical Engineering, Chinese Academy of Medical Sciences & Peking Union Medical College, Tianjin, China

**Keywords:** traditional Chinese exercises, evidence map, randomized controlled trials, systematic reviews, Tai Chi

## Abstract

**Objective:**

This study aimed to assess and visually depict the clinical evidence landscape of traditional Chinese exercises and identify any research gaps and future research needs.

**Methods:**

We comprehensively searched seven Chinese and English databases to identify randomized controlled trials (RCTs) and systematic reviews (SRs) evaluating the effects of traditional Chinese exercises from their inception until May 2023. The quality of evidence was assessed via the GRADE approach, and the research topics, intervention effects, and strength of evidence were graphically displayed.

**Results:**

This evidence map includes 2,017 studies, comprising 1,822 RCTs and 195 SRs. These studies were conducted globally in various countries. Among the traditional Chinese exercises, Tai Chi and Baduanjin have received the most research attention, with a growing number of publications. When traditional Chinese exercises were compared with the control groups, 88.2% of the included SRs reported significantly positive effects, 4.1% reported unclear effects, and 7.7% reported no significant differences. The findings suggested that traditional Chinese exercises could benefit patients with osteoarthritis, osteoporosis, hypertension, coronary heart disease, diabetes, chronic obstructive pulmonary disease, stroke, Parkinson’s disease, anxiety, and depression. However, the overall quality of the evidence was suboptimal, with 11.3% rated as moderate, 45.6% as low, and 43.1% as critically low.

**Conclusion:**

This evidence map visually represents valuable information on traditional Chinese exercises. While most studies have reported significant benefits, the overall quality of evidence is low.

## 1 Introduction

Traditional Chinese exercises consist of Tai Chi, Baduanjin, Liuzijue, Wuqinxi, etc., harmonizing gentle movements with muscle stretching and relaxation, breathing techniques, and mental focus to promote health and well-being and improve medical conditions (1–3). These unique characteristics have contributed to their global recognition and popularity. A large number of studies have reported that traditional Chinese exercises could be beneficial for a wide range of medical conditions in recent years, including cardiovascular diseases (4), respiratory system diseases (5), musculoskeletal system diseases (6), endocrine diseases (7), balance problems (8), etc.

Although previous studies have synthesized studies on traditional Chinese exercises, they have not been comprehensive. An evidence map, focusing specifically on Tai Chi, included relevant systematic reviews and objectively demonstrated the effects of Tai Chi on health outcomes. However, it did not cover other traditional Chinese exercises besides Tai Chi (9). Another evidence map on mind–body exercises included Tai Chi and Baduanjin without other types of traditional Chinese exercises, whereas included other mind–body therapies such as acupressure Shiatsu and Tuiná (10). Moreover, a bibliometric analysis, while objectively showing the publication trends, country and disease distribution of all clinical studies of traditional Chinses exercises, did not present the therapeutic efficacy and quality of evidence (3). Synthesizing the existing research to date, despite the growth of related research, there is still a lack of overviews for the synthesis of all types of traditional Chinese exercises and its clinical evidence. Therefore, one of the primary research interests in this area is the use of a comprehensive approach to highlight what is known and where gaps exist in traditional Chinese exercises.

An evidence map serves as a valuable tool because it constitutes a systematic search of a broad field to identify gaps in knowledge and/or future research needs that present results in a user-friendly format, often a visual figure or graph (11). It draws upon various sources of clinical evidence to facilitate a systematic and visual analysis of a specific topic, covering the volume, findings, and quality of evidence (12). This makes it a valuable resource for healthcare practitioners, providing an easily accessible source to support clinical decision-making processes.

We constructed an evidence map to provide a visual overview of the available evidence on traditional Chinese exercises for different diseases and conditions, facilitate interpretation of the results, and identify future study directions.

## 2 Methods

We developed this evidence map on the basis of *Evidence & Gap Maps: A tool for promoting evidence-informed policy and strategic research agendas* (13). The study was reported in compliance with the *PRISMA Extension for Scoping Reviews (PRISMA-ScR): Checklist and Explanation* (14).

### 2.1 Eligibility criteria

Randomized clinical trials (RCTs) and systematic reviews (SRs) focusing on traditional Chinese exercises that included adult participants with any conditions and without language limitations were included. We excluded interventions of studies involving the combination of multiple types of traditional Chinese exercises or those combined with other interventions.

### 2.2 Information source, literature search, and data extraction

The PubMed, Embase, Cochrane Library, Chinese Biomedical Literature Database (Sino-Med), China National Knowledge Infrastructure (CNKI), Chinese Scientific Journal Database (VIP), and Chinese Academic Conference Papers Database and Chinese Dissertation Database (Wan Fang) were searched for RCTs and SRs on traditional Chinese exercises from inception to May 2023. The search strategy is shown in Supplementary Table S1.

Two researchers (Y Yu and MR Wu) screened the literature and extracted the data independently. For each of the included RCTs, we extracted the following data to synthesize narratively: year, type of interventions and diseases/symptoms. The data items of SRs for the evidence map included the characteristics of the studies, such as author, year, journal, country, type of interventions, comparison, diseases/symptoms, outcomes, and the number of RCTs included in SRs. We also extracted the effect estimates for the primary outcomes and key results. Disagreements were resolved by consensus, and a third researcher (LH Yang) was consulted when necessary.

### 2.3 Quality assessment, analysis and evidence map production

Only the effect estimates and quality of evidence for SRs were depicted on the evidence map, as they were considered the highest level of evidence.

#### 2.3.1 Quality assessment

The Grading of Recommendations, Assessment, Development, and Evaluations (GRADE) was used by two researchers (Y Yu and MR Wu) to assess the quality and certainty of evidence for the SRs. The quality of evidence for the primary outcome was rated as high, moderate, low, and critically low based on five domains: limitations in the study design (risk of bias), inconsistency of results, indirectness of evidence, imprecision of the results, and publication bias. In cases where there is inconsistency in the quality assessments among the primary outcomes, the results with the lowest quality should be selected as the overall quality assessment for the systematic review. If there was any uncertainty, a third researcher (LH Yang) was consulted.

#### 2.3.2 Effect estimates

The effects of traditional Chinese exercises were evaluated based on the results of the primary outcomes reported in the SRs. The estimated effects were classified as “positive effect” when the results of the included SRs on the topic were all significantly positive, “unclear effect” when the results were mixed findings, and “no effect” when the results were not significantly different between groups.

Microsoft Excel 2021 was used for analysis and graph production.

## 3 Results

### 3.1 Data sources and study characteristics

**Figure 1:**
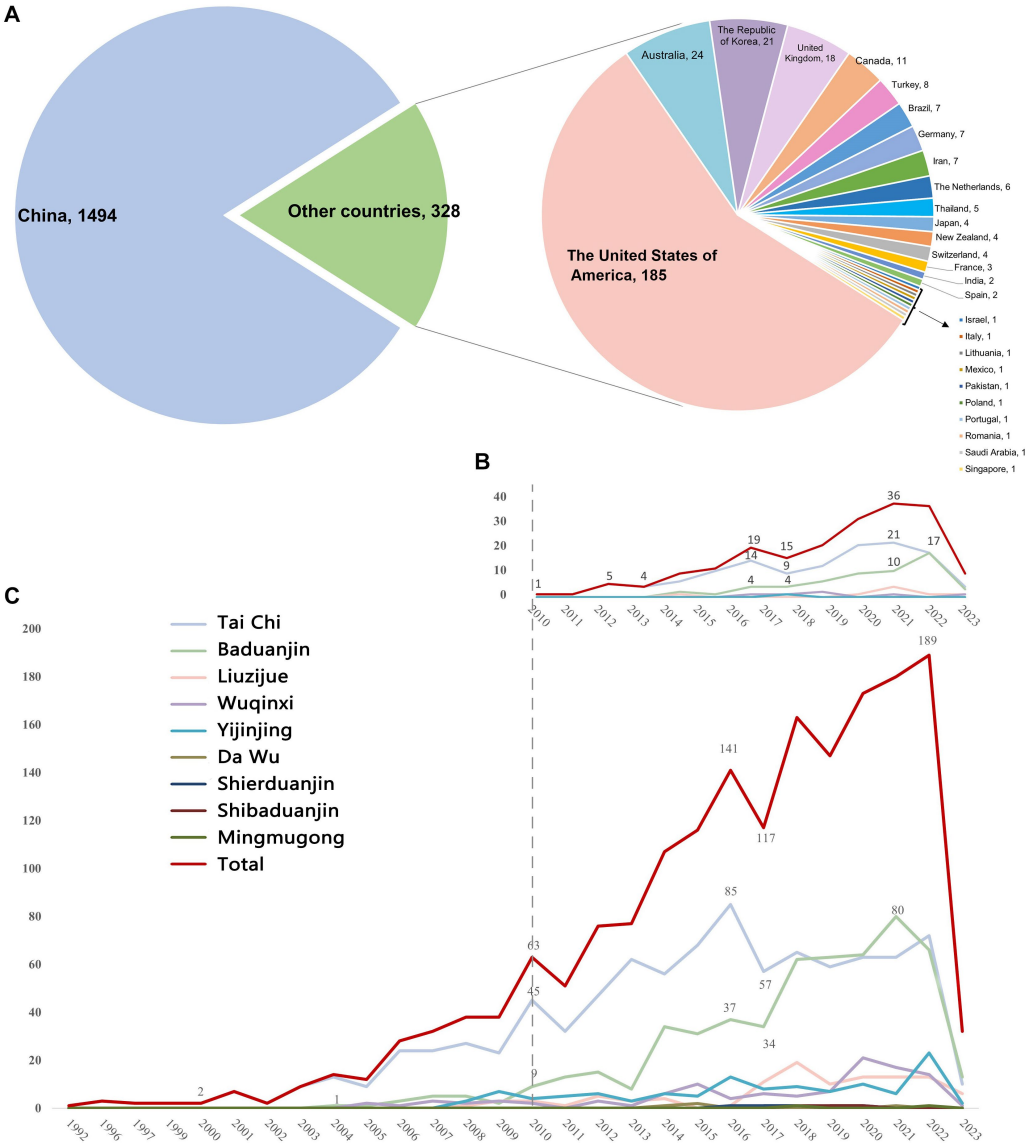
Country distribution and annual trend of publications on traditional Chinese exercises. **(A)** Country distribution of publications on traditional Chinese exercises. **(B)** Annual trend of publications of SRs on traditional Chinese exercises. **(C)** Annual trend of publications of RCTs on traditional Chinese exercises.

The search initially yielded 21,458 citations, and 2,017 eligible studies were ultimately identified, including 1,822 RCTs and 195 SRs. The study identification, selection process and list of the included SRs were illustrated in Supplementary Figure S1 (PRISMA flow diagram) and Supplementary Table S2. Among these studies, nine types of traditional Chinese exercise were reported, with 719 published in English and the others in Chinese. RCTs have been conducted in various countries, including China (81.9%), the United States of America (10.2%), Australia (1.3%), the Republic of Korea (1.2%), and the United Kingdom (1.0%) (Figure 1). The detailed characteristics of the included RCTs and SRs are specified in Supplementary Tables S3, S4, respectively.

### 3.2 Annual trend of the publications

The first published RCT, which evaluated the effects of Tai Chi on mental and emotional stress, was published in 1992 (15). The publication of SRs began in 2010 with research on the effects of Tai Chi for older adults on fall prevention and balance function (16).

Since 2000, the number of RCTs has gradually increased annually, with a higher growth rate between 2011 and 2022 than between 2000 and 2010. Research on RCTs peaked in 2022, with 189 studies published. Since 2010, the number of SRs has increased, with a peak of 36 studies published in 2021 (Figure 1).

### 3.3 Interventions

**Figure 2:**
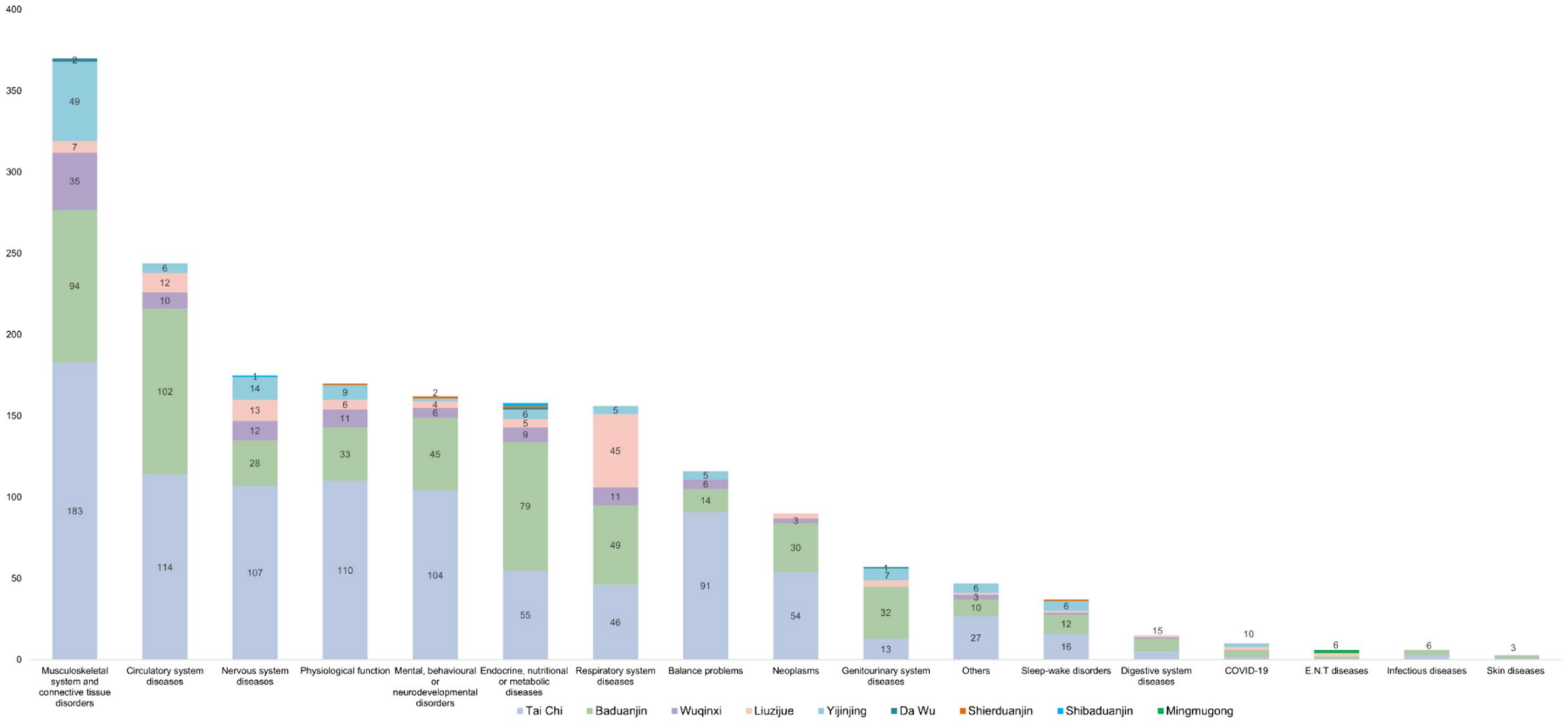
The distribution of interventions of traditional Chinese exercises (RCTs). Other indicators include fatigue, quality of life, inflammation, and biochemical indices.

Tai Chi was the most frequently assessed intervention, accounting for 51.2% of the RCTs, followed by Baduanjin (30%), Yijinjing (6.4%), Wuqinxi (5.9%) and Liuzijue (5.8%), the rest were Dawu (0.22%) (17), Shierduanjin (0.22%) (18), Shibaduanjin (0.16%) (19), and Mingmugong (0.11%) (20), (Figure 2; Supplementary Figure S2 (SRs)).

### 3.4 Diseases and conditions

**Figure 3:**
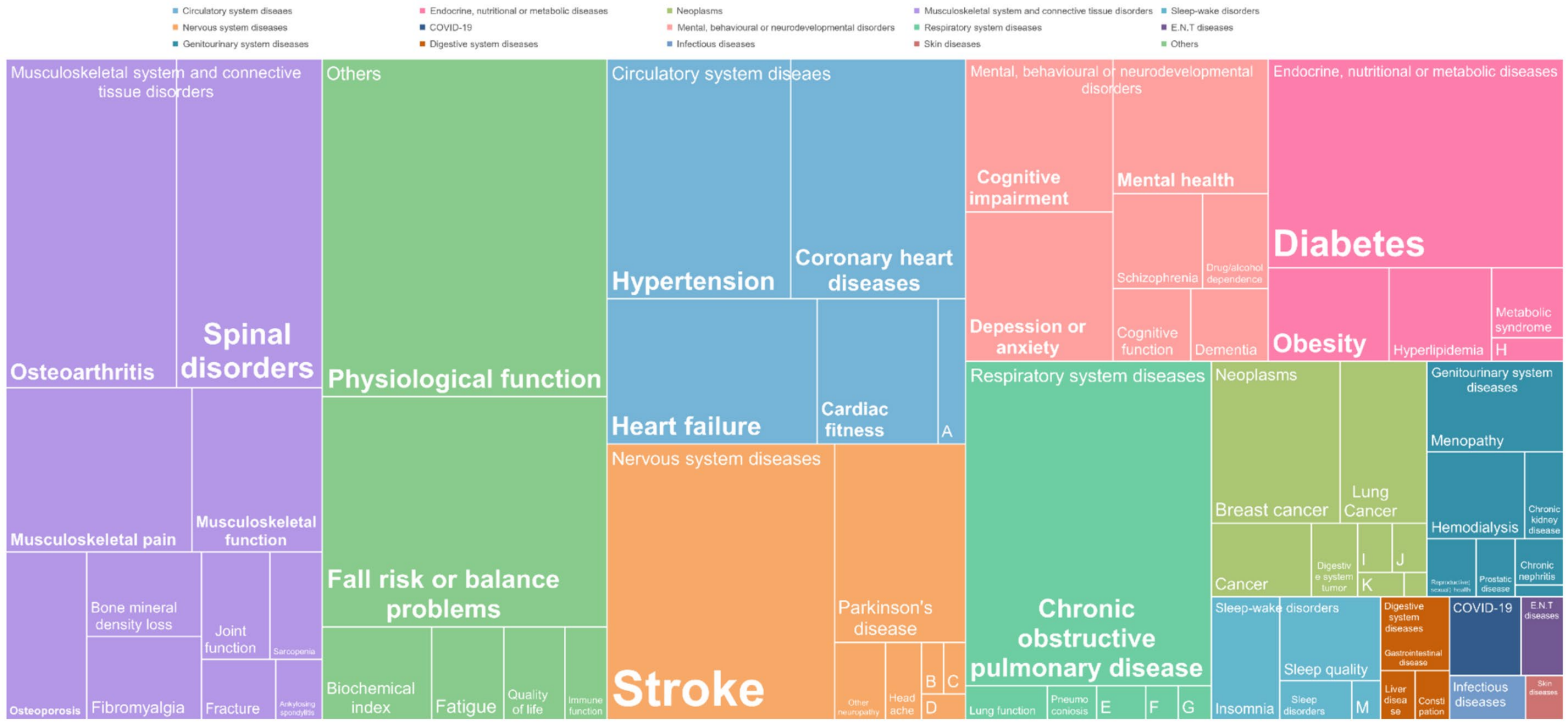
Distribution of diseases and conditions studied in RCTs evaluating the effects of traditional Chinese exercises. A: Cerebral vascular disorders; B: Chronic fatigue syndrome; C: Neurasthenia; D: Multiple sclerosis; E: Mechanical ventilation; F: Bronchial asthma; G: Cystic fibrosis; H: Polycystic ovary syndrome; I: Cancer-related fatigue; J: Prostate cancer; K: Nasopharyngeal carcinoma; L: Non-Hodgkin lymphoma; M: Obstructive sleep apnea syndrome; N: Chronic pelvic inflammatory disease.

The diseases and conditions studied in the SRs and RCTs were generally consistent (Figure 3; Supplementary Figure S3). 79 diseases and conditions classified by the ICD-11 were involved, including musculoskeletal system and connective tissue disorders (20.3%), circulatory system diseases (13.4%), nervous system diseases (9.6%), mental, behavioral, or neurodevelopmental disorders (8.9%), endocrine, nutritional, and metabolic diseases (8.7%), and respiratory system diseases (8.6%).

### 3.5 Evidence map

**Figure 4:**
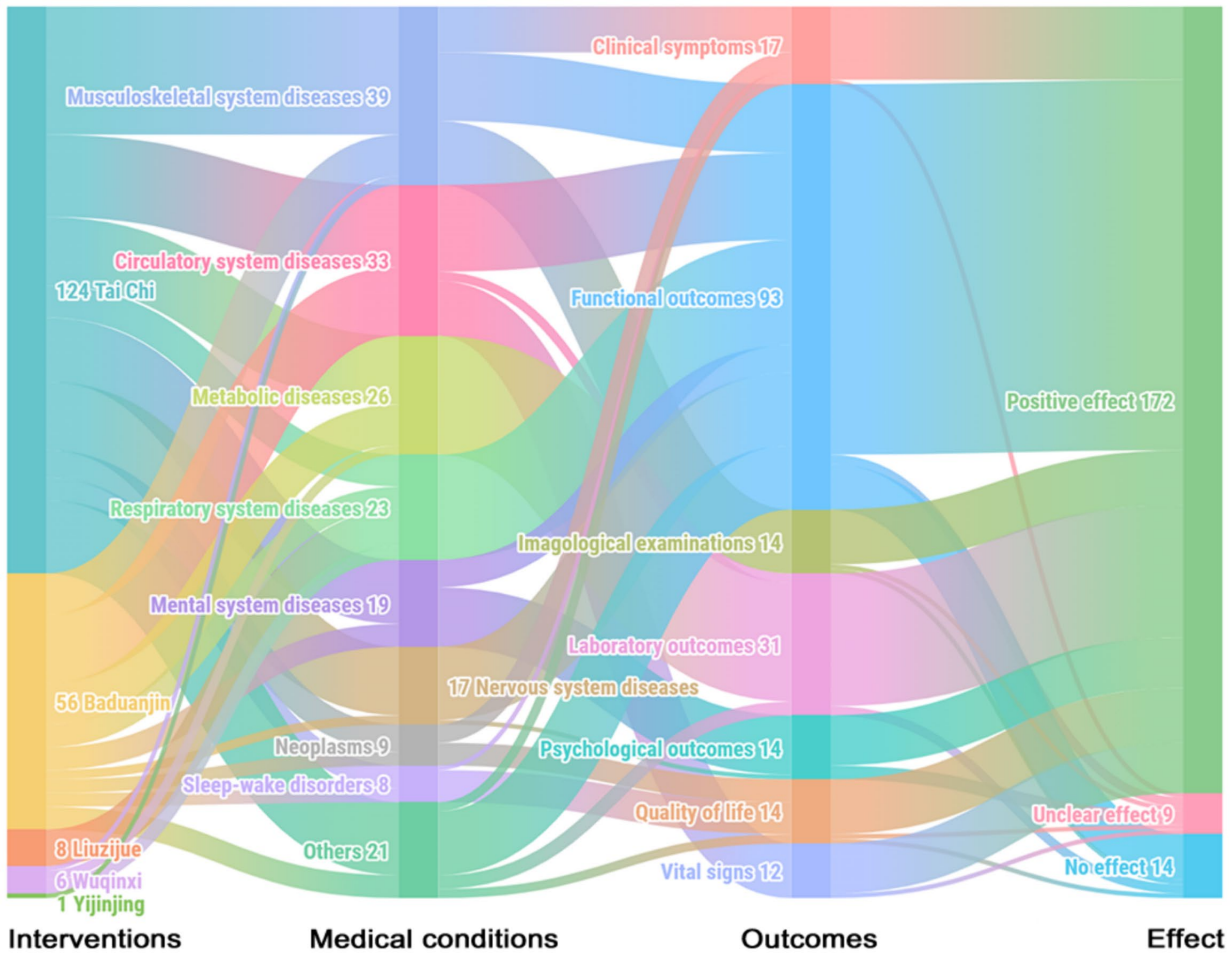
Evidence map of systematic reviews evaluating the effects of traditional Chinese exercises. Clinical symptoms include chronic pain and fatigue. Functional outcomes include cardiopulmonary function, balance function, and cognitive function. Imagological examinations include bone density examinations. Laboratory outcomes include blood lipids, blood glucose, and inflammatory factors. Psychological outcomes include anxiety, depression, etc. Quality of life includes overall quality of life and sleep quality. Vital signs include blood pressure.

**Figure 5:**
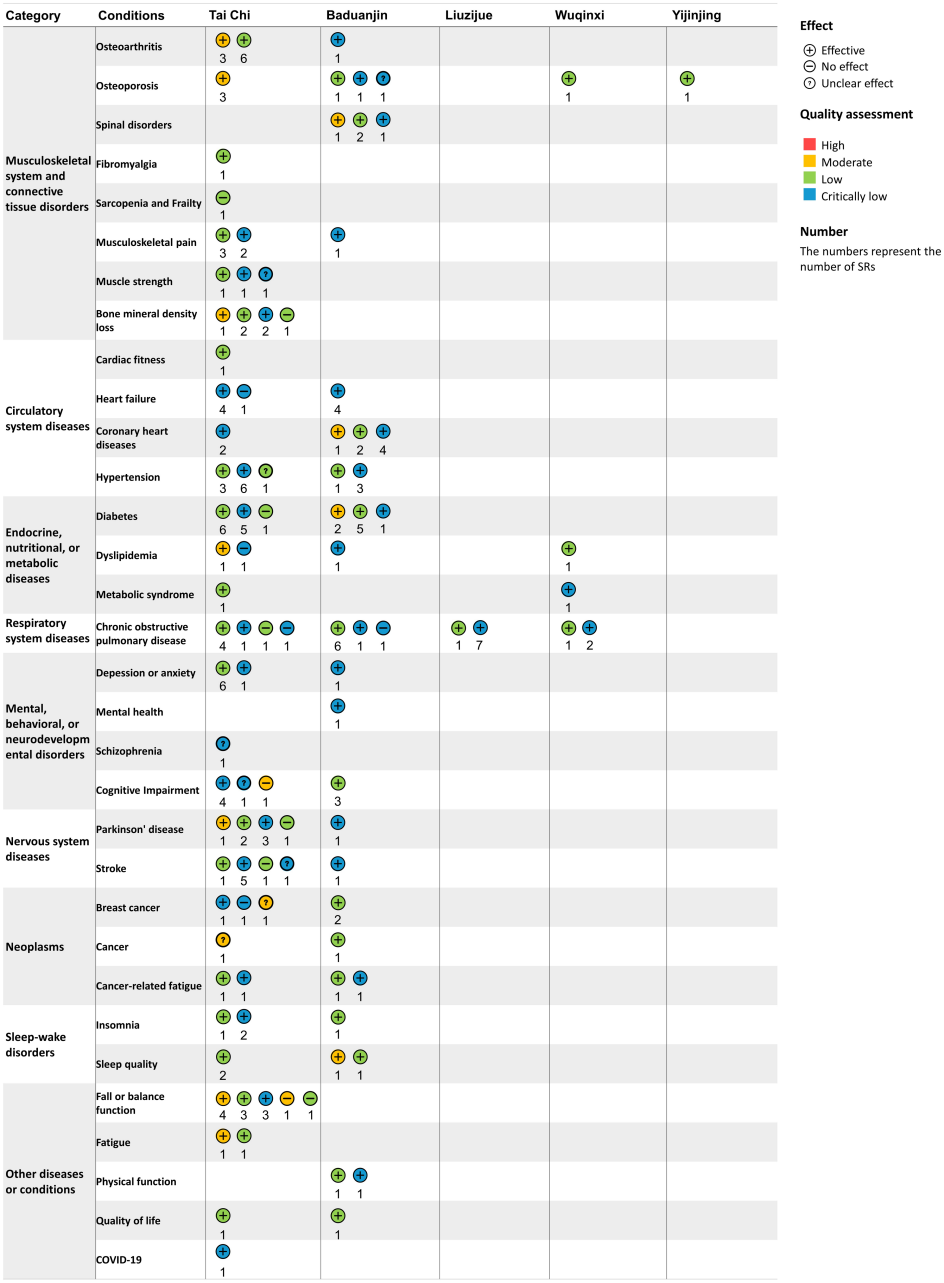
Conditions, quality assessment and effects of interventions in systematic reviews included in the traditional Chinese exercise evidence map. The horizontal axis represents types of traditional Chinese exercise, and the vertical axis represents different diseases and conditions. The level of GRADE was categorized as critically low, low, moderate, or high. No effect: There was no significant difference between traditional Chinese exercises and controls. Unclear effects: The results revealed mixed findings. Positive effect: effect estimates of traditional Chinese exercises are significantly positive. The numbers represent the number of SRs.

Among the 195 included SRs, 88.2% (*n* = 172) reported that traditional Chinese exercises were more effective than the control methods in terms of outcomes, which included clinical symptoms, functional outcomes, imagological examinations, laboratory outcomes, psychological outcomes, quality of life and vital signs. Only 4.6% (*n* = 9) showed unclear effects, and 7.2% (*n* = 14) reported no significant difference between the traditional Chinese exercise group and the control group, mainly in terms of functional outcomes. Studies have shown unclear effects, often due to the use of various primary outcomes in SRs. Regarding the quality of the evidence for SRs, 11.3% were assessed as moderate quality, 45.6% as low quality, and 43.1% as critically low quality according to GRADE. The evidence map of all the diseases and conditions is shown in Figures 4, 5. Further detailed evidence organized by conditions is presented in the following paragraphs, and their evidence maps are available in Supplementary Table S5, offering a comprehensive overview of our findings.

#### 3.5.1 Musculoskeletal system and connective tissue disorders

Tai Chi was found to improve bone mineral density for osteoporosis (moderate-quality evidence) and alleviate pain, stiffness and function for osteoarthritis, assessed by the Western Ontario and McMaster Universities Arthritis Index (low-to moderate-quality evidence). There were also potential benefits of Tai Chi for musculoskeletal pain (critically low-to low-quality evidence) and fibromyalgia according to the fibromyalgia impact questionnaire (low-quality evidence). Baduanjin was found to be effective for treating spinal disorders in terms of the response rate (critically low to moderate quality) and beneficial for relieving musculoskeletal pain (critically low-quality evidence). Wuqinxi and Yijinjing reportedly have positive effects on osteoarthritis (low-quality evidence). However, the effects of Tai Chi on muscle strength and bone mineral density loss, as well as Baduanjin on osteoporosis, are unclear. Low-quality evidence revealed that Tai Chi did not significantly improve muscle mass or other sarcopenia-related outcomes (Supplementary Table S5).

#### 3.5.2 Circulatory system diseases

Tai Chi was found to be beneficial for the cardiorespiratory fitness of older adult individuals by increasing the maximum oxygen consumption rate and leading to an overall reduction in heart rate (low-quality evidence). It also improved cardiopulmonary exercise capacity in patients with coronary heart disease, as indicated by the 6 min walk test (6MWT) (critically low-quality evidence). Baduanjin showed clinical benefits in reducing the frequency of angina pectoris and promoting cardiopulmonary health in patients with coronary heart disease (critically low-to moderate-quality evidence), as well as in reducing the blood pressure of patients with hypertension (critically low-quality evidence). It had also been shown to improve cardiopulmonary health and quality of life in patients with heart failure (critically low-quality evidence). However, the effects of Tai Chi on hypertension (critically low-to low-quality evidence) and heart failure (critically low-quality evidence) were inconclusive (Supplementary Table S5).

#### 3.5.3 Endocrine, nutritional, or metabolic diseases

Baduanjin was found to have positive effects on lowering fasting blood glucose and glycosylated hemoglobin in patients with diabetes (critically low-to moderate-quality evidence) and regulating triglycerides, total cholesterol, and high-density and low-density lipoprotein cholesterol in patients with dyslipidemia (critically low-quality evidence). Wuqinxi was beneficial for improving lipid outcomes in patients with dyslipidemia (critically low-to low-quality evidence), decreasing blood pressure and glucose, and regulating lipids in patients with metabolic syndrome (critically low-quality evidence). Tai Chi was also effective in ameliorating metabolic syndrome (low-quality evidence). Nevertheless, the effects of diabetes (critically low-to low-quality evidence) and dyslipidemia (critically low-to low-quality evidence) were unclear (Supplementary Table S5).

#### 3.5.4 Respiratory system diseases

Liuzijue and Wuqinxi reported positive effects on chronic obstructive pulmonary disease (COPD) (critically low-to low-quality evidence) in terms of improved motor function and pulmonary function assessed by 6MWT, forced expiratory volume in the first second (FEV1), forced vital capacity (FVC) and the ratio of forced expiratory volume in the first second to forced vital capacity (FEV1/FVC). The effects of Tai Chi and Baduanjin on COPD were unclear (critically low-to low-quality evidence) (Supplementary Table S5).

#### 3.5.5 Mental, behavioral, or neurodevelopmental disorders

Both Tai Chi and Baduanjin have positive effects on anxiety, depression and mental health (critically low-to low-quality evidence). Baduanjin was beneficial for cognitive impairment (low-quality evidence) in global cognitive function, although the effects of Tai Chi on cognitive impairment were inconclusive (critically low-to moderate-quality evidence). Tai Chi was effective in reducing negative symptoms but did not have significant effects on positive symptoms of schizophrenia (critically low quality) (Supplementary Table S5).

#### 3.5.6 Nervous system diseases

Baduanjin demonstrated clinical benefits for patients with Parkinson’s disease (critically low-quality evidence), as evidenced by improvements in total UPDRS-III scores for daily living, motor function and motor complications, as well as enhanced balance function in stroke patients (critically low-quality evidence). However, the effects of Tai Chi on balance function in patients with Parkinson’s disease (critically low-to moderate-quality evidence) and stroke were unclear (critically low-to moderate-quality evidence) (Supplementary Table S5).

#### 3.5.7 Neoplasms

Baduanjin could improve quality of life and alleviate fatigue in cancer patients (low-quality evidence), whereas the effects of Tai Chi on these outcomes in cancer patients were inconclusive (critically low-to moderate-quality evidence) (Supplementary Table S5).

#### 3.5.8 Sleep–wake disorders

Both Tai Chi and Baduanjin had positive effects on sleep quality and insomnia, despite their low or critically low quality (Supplementary Table S5).

#### 3.5.9 Other diseases or conditions

Tai Chi could improve symptoms of fatigue (moderate-quality evidence) and quality of life (low-quality evidence). Clinical benefits were also observed in outcomes related to inflammation, such as C-reactive protein and TNF-alpha, in COVID-19 patients (critically low quality). Baduanjin had positive effects on balance function (critically low-to low-quality evidence), physical function (critically low-to low-quality evidence) and quality of life (low-quality evidence). However, the effects of Tai Chi on balance function and fall risk remained inconclusive (critically low-to low-quality evidence) (Supplementary Table S5).

## 4 Discussion

### 4.1 Publication of studies

This evidence map provides a broad overview of the clinical evidence on traditional Chinese exercises, drawing from a pool of 2,017 published studies. While the majority of the evidence originated from China, approximately 18% of the studies included in this evidence map were conducted globally. This highlights the widespread interest and collaborative effort toward advancing knowledge in this field. Policies and institutional support have fostered the development of traditional Chinese exercises. In 2000, the General Administration of Sport of China issued the first management document on traditional Chinese exercises, known as *Interim Measures for the Management of Fitness Qigong,* followed by the establishment of the Qigong Administrative Center, which increased researchers’ attention and interest in this field and led to rapid growth in related studies.

### 4.2 Effectiveness of traditional Chinese exercises and their potential mechanisms

Many studies have shown that Tai Chi is particularly beneficial for conditions such as osteoarthritis, osteoporosis, coronary heart disease, anxiety and depression, and sleep–wake disorders. The mechanisms of Tai Chi for these conditions can be attributed to four points. First, compelling evidence has demonstrated that Tai Chi can enhance motor coordination and capacity, improve gait and postural stability, and increase muscle strength and trunk flexibility (21–25). Second, Tai Chi can stimulate the excitability of parasympathetic nerves, enhance vascular compliance, induce vasodilation, and reduce oxidative stress and inflammation to prevent elastic artery stiffness and endothelial dysfunction, thereby mitigating the risk of coronary heart diseases and other circulatory system diseases (26–28). Third, studies have shown that Tai Chi can improve sleep quality, which is consistent with previous studies that exercise can promote deeper and more restorative sleep, which in turn can reduce C-reactive protein levels in the body, thereby improving systemic inflammatory responses (29, 30). Fourth, Tai Chi has the capacity to augment autonomic regulation, strengthen parasympathetic nerves, stimulate the secretion of norepinephrine and dopamine, and extend telomeres while increasing telomerase activity. These functions contribute to alleviating negative psychological symptoms in individuals (31–34).

Baduanjin has demonstrated positive effects on a range of diseases and conditions, including dyslipidemia, diabetes, heart failure, coronary heart disease, musculoskeletal pain, spinal disorders, Parkinson’s disease, stroke, balance problems, cognitive impairment, cancer-related fatigue, and sleep–wake disorders. These diverse benefits of Baduanjin can be attributed to four main mechanisms. First, as a low-intensity aerobic exercise, Baduanjin has been shown to modulate the expression of mRNAs, long noncoding RNAs (lncRNAs), and circular RNAs (circRNAs) (35), which in turn facilitates increased muscle absorption, transportation, and utilization of lipids and glucose, thereby significantly regulating overall metabolic function (36–39). Second, Baduanjin can reduce myocardial oxygen consumption and improve cardiorespiratory function (40–42). Third, the combination of concentric and eccentric contractions during Baduanjin training is more effective in improving muscle strength, mobility and balance function (43). Fourth, the interaction between exercise, nutrition, and sleep is crucial for enhancing cognitive function and mitigating sleep disturbances (44, 45). Regular practice of Baduanjin is known to improve sleep quality and cognitive performance, while a balanced diet rich in essential nutrients, such as antioxidants and live microbes, supports neuroplasticity and brain function (46, 47). Thus, future research could delve deeper into these interactions, combining traditional Chinese exercises with a nutritious diet, to develop comprehensive interventions that optimize cognitive health and sleep quality, particularly for those at risk.

Liuzijue is particularly specialized for treating COPD (48, 49). Studies have shown that the distinctive breathing techniques of Liuzijue effectively mobilize respiratory muscles (50), increase the range of diaphragmatic movement, and enhance the strength of respiratory muscles (5). These benefits include the extension of expiratory time, increased respiratory depth, improved airway pressure, and increased pulmonary ventilation function (5, 51).

Wuqinxi has shown clinical benefits for treating osteoarthritis, metabolic syndrome, and COPD, whereas Yijinjing has been shown to positively impact osteoarthritis. The bird-like movements in Wuqinxi are conducive to maintaining the continuity and duration of breathing, thereby improving pulmonary ventilation (52). Furthermore, both Wuqinxi and Yijinjing contribute to muscle strengthening and maintaining normal bone and joint structures (53–56). Further details on the characteristics and mechanisms of traditional Chinese exercises can be found in Supplementary Table S6.

### 4.3 Implementations for future research

This evidence map demonstrated that traditional Chinese exercises, notably Tai Chi and Baduanjin, improved conditions such as osteoarthritis, musculoskeletal pain, coronary heart disease, anxiety, depression, and insomnia. Some of these improvements have been supported by moderate-certainty evidence, while the quality of the majority of evidence has been rated as low or critically low. The downgrading of the certainty of evidence is attributed primarily to the high risk of bias in the studies, imprecision of the results, and heterogeneity between the studies.

A large proportion of the trials were assessed as having a high risk of bias because of unclear or inadequate random-sequence generation, allocation concealment, and lack of or unclear double-blinding. It was reported that the inadequacy or lack of these elements in RCTs was associated with 7 to 13% exaggeration of the intervention effects, and a lack of blinding had the most significant impact (57). Future RCTs must incorporate adequate random-sequence generation and allocation concealment in their study design. Blinding in a clinical trial involving traditional Chinese exercises can be challenging due to the nature of the intervention, which involves physical movements and interactions between participants and instructors. However, several efforts can be made to reduce bias. Instructors who teach traditional Chinese exercises can be blinded. They can be told that they are teaching a traditional Chinese exercise class without revealing the details of the study, such as the specific group assignments and the study’s hypotheses. It is advisable to use objective outcomes rather than subjective outcomes as the primary outcome whenever possible. This is because objective outcomes are less likely to be influenced by a lack of blinding. Additionally, efforts can be made to reduce the risk of measurement bias by blinding outcome assessors, data managers, and statisticians.

Inadequate sample sizes can lead to imprecise and inconclusive results. Determining the appropriate sample size according to a rational estimation can ensure that the study is adequately powered to detect meaningful differences. Moreover, standardizing the exercise intervention to minimize variability can enhance consistency and improve the precision of the results. This evidence map also encompasses a substantial number of studies on conditions such as osteoporosis, hypertension, heart failure, diabetes, dyslipidemia, cognitive impairment, Parkinson’s disease, stroke, and balance function. Nevertheless, the therapeutic effectiveness of traditional Chinese exercises for these conditions remains unclear, and further research is needed. The inconclusive results may be due to the inappropriateness of trial designs, including participants, differences in interventions, comparators, and outcomes.

Each traditional Chinese exercise type comprises a distinct series of postures and movements, specifically emphasizing different functional improvements. For example, Tai Chi involves more intricate and continuous flowing sequences of postures, emphasizing balance and stability (58). Yijinjing and Wuqinxi specifically target muscle and tendon strength and flexibility (59, 60). Baduanjin is gentler, consisting of only eight simple repetitive exercises, making learning easier (61). Liuzijue is supplemented by body movements and mainly focuses on breathing techniques, such as deep, slow, vocal and reverse abdominal breathing, to exercise the function of respiratory muscles (5, 62). Furthermore, for certain types of exercise, such as Tai Chi, Yijinjing and Wuqinxi, which involve complex movements, including knee bends, squats, and standing up, it is essential to evaluate whether they are suitable for individuals with movement disorders, such as severe cognitive impairment, Parkinson’s disease, and stroke.

### 4.4 Limitations

Several limitations are acknowledged in this evidence map. First, given the large amount of literature on this topic, we only searched English and Chinese literature databases, focusing on RCTs and SRs, and evidence from other sources and lower-grade study designs were ignored. Second, although the included studies provide a very extensive overview of traditional Chinese exercises, only a rough qualitative description of the included RCTs cannot answer more refined questions; future research can focus on a particular clinical condition for qualitative and quantitative synthesis.

## 5 Conclusion

This evidence map summarizes and provides a visual overview of the available evidence hotspots. Most studies reported positive effects on functional outcomes as well as laboratory indices. Tai Chi, Yijinjing, and Wuqinxi have significantly improved patients’ condition, particularly in musculoskeletal disorders. Baduanjin tends to be more effective in endocrine, metabolic, and circulatory diseases. Liuzijue primarily targets COPD treatment. However, more high-quality evidence is warranted to support these findings further.

## Data Availability

The original contributions presented in the study are included in the article/Supplementary material, further inquiries can be directed to the corresponding authors.
