# Supplementary figures and images for: Evidence map of traditional Chinese exercises
Source: Front Public Health. 2024 Sep 18;12:1347201. doi: 10.3389/fpubh.2024.1347201 (PMC11445016; doi:10.3389/fpubh.2024.1347201)

**Supplementary Figure 1**


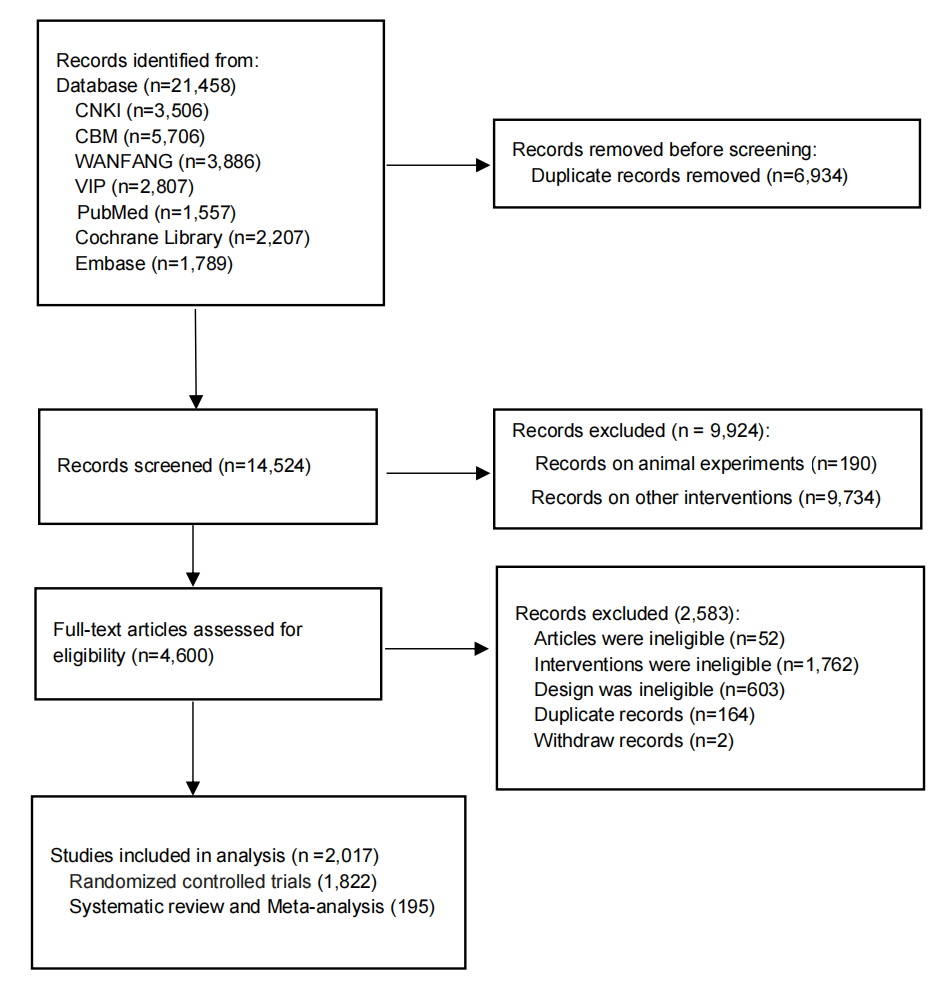


**Supplementary Figure1.** Flowchart of study identification.

Supplement: Supplementary file 1 [file Data_Sheet_1.docx]
