# Supplementary material for: Evidence map of traditional Chinese exercises
Source: Front Public Health. 2024 Sep 18;12:1347201. doi: 10.3389/fpubh.2024.1347201 (PMC11445016; doi:10.3389/fpubh.2024.1347201)
Supplement: Supplementary file 2 [file Data_Sheet_2.docx]

**Supplementary Figure**


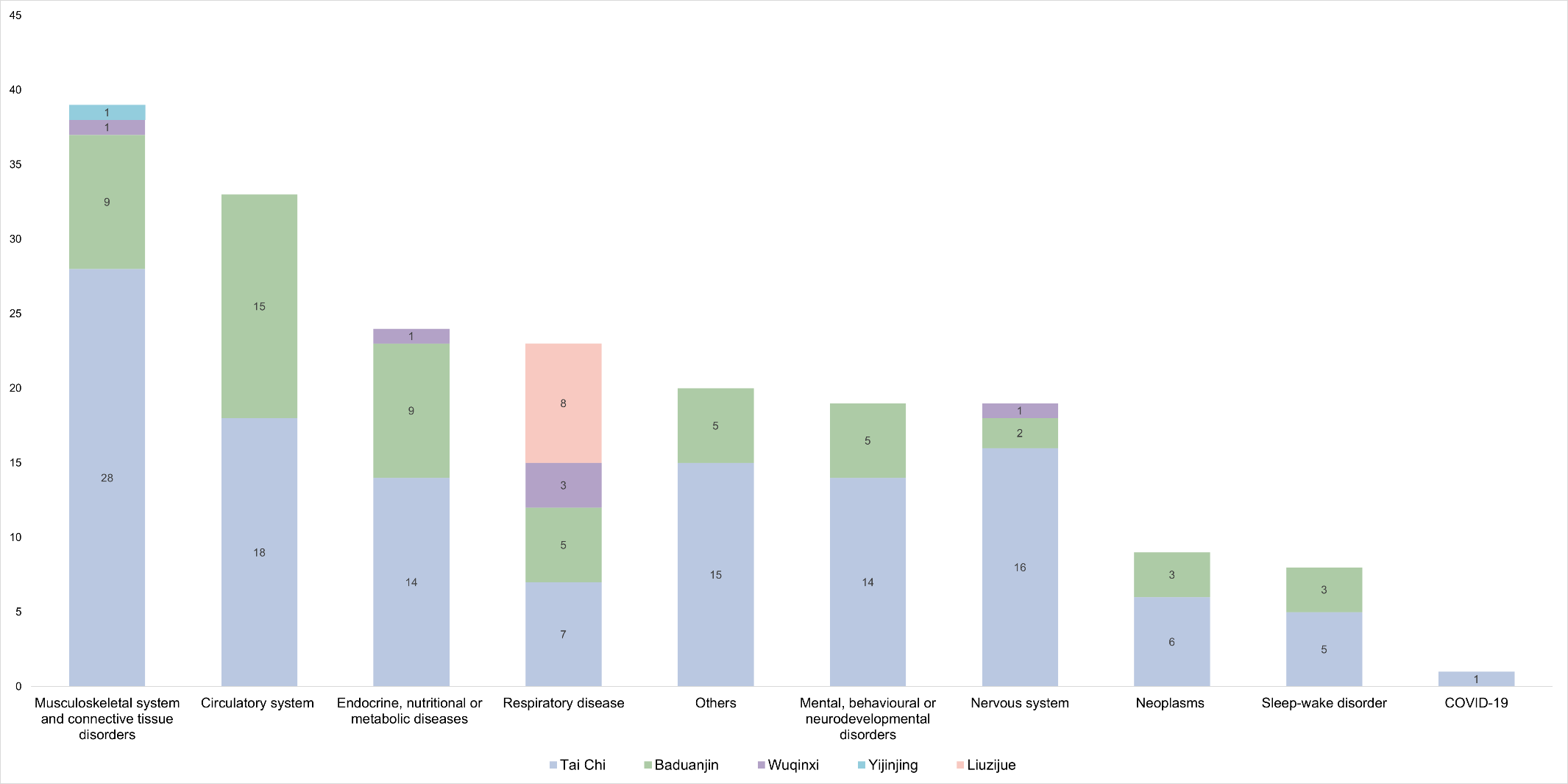


**Supplementary Figure 2.** The distribution of interventions of traditional Chinese exercises (SRs).

**
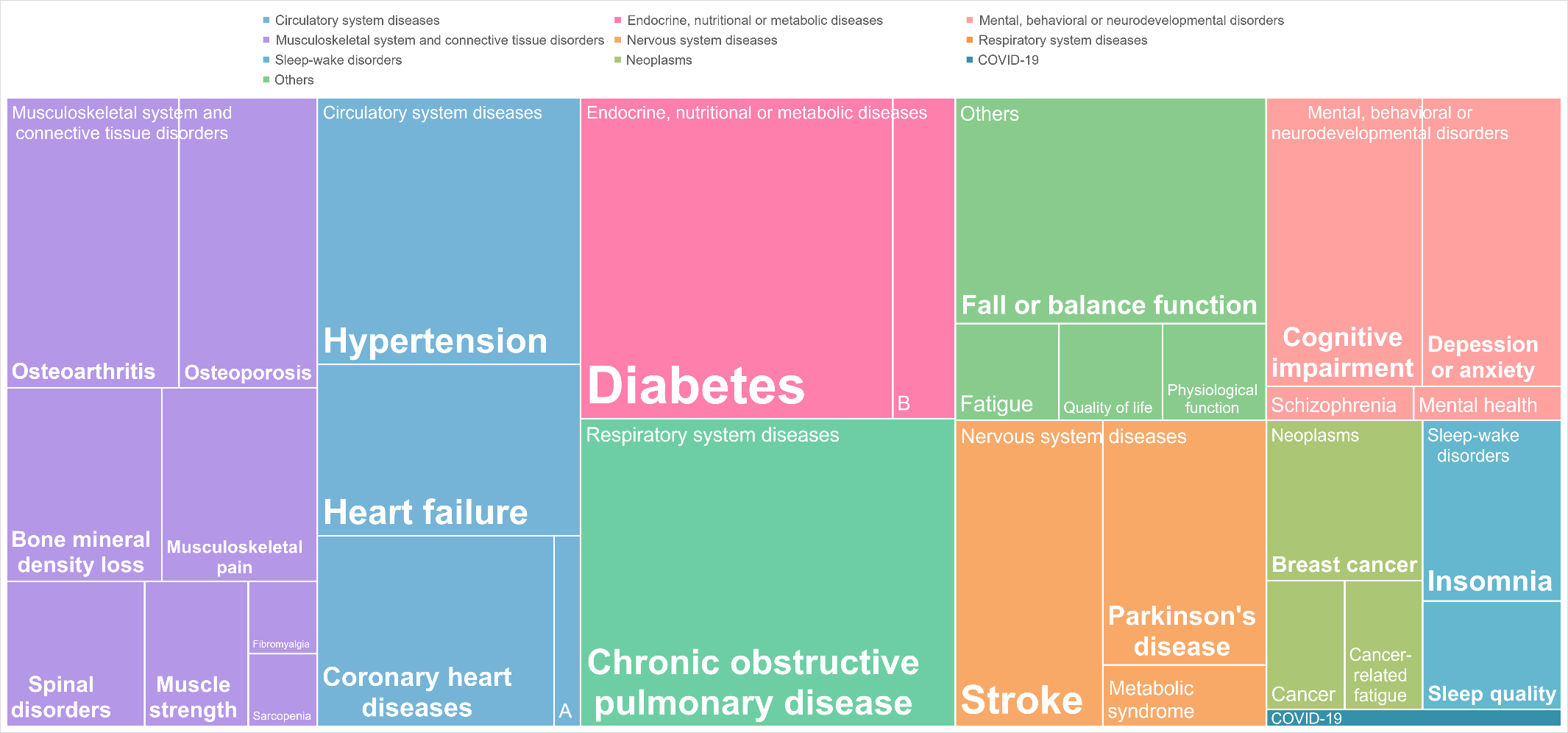
**

**Supplementary Figure 3.** The distribution of diseases/conditions for traditional Chinese exercises (SRs).

Note: A: Cardiac fitness; B: Dyslipidemia
