# Supplementary material for: Evidence map of traditional Chinese exercises
Source: Front Public Health. 2024 Sep 18;12:1347201. doi: 10.3389/fpubh.2024.1347201 (PMC11445016; doi:10.3389/fpubh.2024.1347201)
Supplement: Supplementary file 3 [file Table_1.DOCX]

**Supplementary Table 1: Search strategy**

| PubMed |
| --- |
| ((Dao Yin[Title/Abstract] OR daoyin[Title/Abstract] OR Ba Duan Jin[Title/Abstract] OR Eight Fragments[Title/Abstract] OR eight section brocade[Title/Abstract] OR eight trigrams boxing[Title/Abstract] OR Yi Jin Jing[Title/Abstract] OR yijinjing[Title/Abstract] OR Wu Qin Xi[Title/Abstract] OR wuqinxi[Title/Abstract] OR Liu Zi Jue[Title/Abstract] OR liuzijue[Title/Abstract] OR Tai Chi[Title/Abstract] OR taiji[Title/Abstract] OR taichi[Title/Abstract] OR Tai Ji[Title/Abstract] OR Qi Gong[Title/Abstract] OR qigong[Title/Abstract] OR Ch'i Kung[Title/Abstract] OR Chi gung[Title/Abstract] OR Qi Training[Title/Abstract] OR Gong Fa[Title/Abstract] OR gongfa[Title/Abstract] OR Shi Er Duan Jin[Title/Abstract] OR Da Wu[Title/Abstract])) AND ((randomized controlled trial[Publication Type]) OR (randomized[Title/Abstract]) OR (systematic review[Title/Abstract]) OR (meta analysis[Title/Abstract])) |
| EMBASE |
| #1 'dao yin':ab,ti OR 'qi gong':ab,ti OR qigong:ab,ti OR gongfa:ab,ti OR daoyin:ab,ti OR 'chi gung':ab,ti OR wuqinxi:ab,ti OR baduanjin:ab,ti OR yijinjing:ab,ti OR 'ba duan jin':ab,ti OR 'wu qin xi':ab,ti OR 'yi jin jing':ab,ti OR liuzijue:ab,ti OR 'tai chi':ab,ti OR taichi:ab,ti OR taiji:ab,ti OR 'tai ji':ab,ti OR 'chi kung':ab,ti OR 'eight section brocade':ab,ti OR 'eight fragments':ab,ti OR 'eight trigrams boxing':ab,ti  #2 randomized:ab,ti OR 'randomized controlled trial':ab,ti OR 'systematic review':ab,ti OR 'meta analysis':ab,ti  #3 #1 and #2 |
| Cochrane Library |
| #1 dao yin OR Dao Yin OR Qi Gong OR qigong OR qi training OR chi kung OR gongfa OR Ba Duan Jin OR badunajin OR eight fragments OR eight section brocade OR eight trigrams boxing OR wuqinxi OR Wu Qin Xi OR Yi Jin Jing OR yijinjing OR Tai Chi OR taiji OR taichi OR Tai Ji OR liuzijue OR da wu OR Liu Zi Jue OR Shi Er Duan Jin  #2 randomized OR randomly OR randomized controlled trial OR systematic review OR meta analysis  #3 #1 and #2 |
| CBM (Chinese database) |
| 高级检索(非智能检索)  ( "导引"[常用字段] OR "功法"[常用字段] OR "气功"[常用字段] OR "八段锦"[常用字段] OR "五禽戏"[常用字段] OR "易筋经"[常用字段] OR "六字诀"[常用字段] OR "十二段锦"[常用字段] OR "大舞"[常用字段] OR "太极"[常用字段]) AND( "随机"[常用字段] OR "随机对照试验"[常用字段] OR "系统评价"[常用字段] OR "meta分析"[常用字段]) |
| CNKI (Chinese database) |
| 高级检索  篇关摘：（导引 + 八段锦 + 易筋经 + 五禽戏 + 功法 + 气功 + 六字诀 + 十二段锦 + 太极 + 大舞（精确））AND（篇关摘：随机 + 随机对照试验 + 系统评价 + meta分析（精确）） |
| Wan Fang (Chinese database) |
| (题名或关键词:(导引 OR 气功 OR 功法 OR 五禽戏 OR 八段锦 OR 易筋经 OR 太极 OR 十二段锦 OR 大舞 OR 六字诀)) AND (题名或关键词：(随机 OR 随机对照试验 OR 系统评价)) |
| VIP (Chinese database) |
| 专业检索  M=(导引 or 气功 or 功法 or 五禽戏 or 八段锦 or 易筋经 or 太极 or 十二段锦 or 大舞 or 六字诀) AND (M=(随机对照试验 OR 随机 OR 系统评价 OR meta分析) OR R=(随机对照试验 OR 随机 OR 系统评价 OR meta分析)) |
