# Supplementary material for: Evidence map of traditional Chinese exercises
Source: Front Public Health. 2024 Sep 18;12:1347201. doi: 10.3389/fpubh.2024.1347201 (PMC11445016; doi:10.3389/fpubh.2024.1347201)
Supplement: Supplementary file 4 [file Table_2.docx]

**Supplementary Table 2. The list of included SRs**

| 1. Alenazi AM, Alshehri M, Hoover JC, et al. The effect of tai chi exercise on lipid profiles. A systematic review and meta-analysis of randomized clinical trials. Cardiopulmonary Physical Therapy Journal 2018;29:32.  2. Cai Q, Cai SB, Chen JK, et al. Tai chi for anxiety and depression symptoms in cancer, stroke, heart failure, and chronic obstructive pulmonary disease: A systematic review and meta-analysis. Complement Ther Clin Pract 2022;46:101510.  3. Cai Y, Liu X, Zhao A, et al. Effects of tai chi on health outcomes in patients with type 2 diabetes mellitus: A systematic review and meta-analysis. Journal of Traditional Chinese Medical Sciences 2022;9:108-20.  4. Cai Z, Jiang W, Yin J, Chen Z, Wang J, Wang X. Effects of tai chi chuan on cognitive function in older adults with cognitive impairment: A systematic and meta-analytic review. Evid Based Complement Alternat Med 2020;2020:6683302.  5. Cao A, Feng F, Zhang L, Zhou X. Baduanjin exercise for chronic obstructive pulmonary disease: An updated systematic review and meta-analysis. Clinical rehabilitation 2020;34:1004-13.  6. Chang WD, Chen S, Lee CL, Lin HY, Lai PT. The effects of tai chi chuan on improving mind-body health for knee osteoarthritis patients: A systematic review and meta-analysis. Evid Based Complement Alternat Med 2016;2016:1813979.  7. Chao M, Wang C, Dong X, Ding M. The effects of tai chi on type 2 diabetes mellitus: A meta-analysis. J Diabetes Res 2018;2018:7350567.  8. Chau JPC, Leung LYL, Liu X, et al. Effects of tai chi on health outcomes among community-dwelling adults with or at risk of metabolic syndrome: A systematic review. Complement Ther Clin Pract 2021;44:101445.  9. Cheng CA, Chiu YW, Wu D, Kuan YC, Chen SN, Tam KW. Effectiveness of tai chi on fibromyalgia patients: A meta-analysis of randomized controlled trials. Complement Ther Med 2019;46:1-8.  10. Chi I, Jordan-Marsh M, Guo M, Xie B, Bai Z. Tai chi and reduction of depressive symptoms for older adults: A meta-analysis of randomized trials. Geriatr Gerontol Int 2013;13:3-12.  11. Dong X, Ding M, Yi X. Meta-analysis of randomized controlled trials of the effects of tai chi on blood pressure. Evid Based Complement Alternat Med 2020;2020:8503047.  12. Du S, Dong J, Zhang H, et al. Taichi exercise for self-rated sleep quality in older people: A systematic review and meta-analysis. Int J Nurs Stud 2015;52:368-79.  13. Du Z, Zhang X, Qin H, Wang R, Bai Y, Yao X. Meta analysis on the effect of taijiquan on improving negative psychological symptoms of college students and the optimal dose. Front Public Health 2022;10:1032266.  14. Gao P, Tang F, Liu W, He K, Mo Y. Effect of liuzijue qigong on patients with stable chronic obstructive pulmonary disease: A systematic review and meta-analysis. Medicine (Baltimore) 2021;100:e27344.  15. Gong X, Rong G, Wang Z, Zhang A, Li X, Wang L. Baduanjin exercise for patients with breast cancer: A systematic review and meta-analysis. Complementary Therapies in Medicine 2022;71.  16. Gu Q, Wu SJ, Zheng Y, et al. Tai chi exercise for patients with chronic heart failure: A meta-analysis of randomized controlled trials. Am J Phys Med Rehabil 2017;96:706-16.  17. Guan Y, Hao Y, Guan Y, Wang H. Effects of tai chi on essential hypertension and related risk factors: A meta-analysis of randomized controlled trials. J Rehabil Med 2020;52:jrm00057.  18. Guo C, Xiang G, Xie L, et al. Effects of tai chi training on the physical and mental health status in patients with chronic obstructive pulmonary disease: A systematic review and meta-analysis. J Thorac Dis 2020;12:504-21.  19. Jiabao G, Yi Z. Effects of tai chi on cardiopulmonary function in patients with chronic obstructive pulmonary disease: A systematic review and meta-analysis. Physiotherapy (United Kingdom) 2015;101:eS681-eS2.  20. Guo L, Liu Z, Yuan W. The effect of baduanjin on the balancing ability of older adults: A systematic review and meta-analysis. Frontiers in Medicine 2022;9.  21. Guo S, Xu Y, Qin J, et al. Effect of tai chi on glycaemic control, lipid metabolism and body composition in adults with type 2 diabetes: A meta-analysis and systematic review. J Rehabil Med 2021;53:jrm00165.  22. Hall A, Copsey B, Richmond H, et al. Effectiveness of tai chi for chronic musculoskeletal pain conditions: Updated systematic review and meta-analysis. Phys Ther 2017;97:227-38.  23. Han D, Cheng J, Qu J, et al. Effectiveness of taijiquan in treating insomnia: A systematic review and meta-analysis of randomized controlled studies. Front Psychiatry 2022;13:892453.  24. Hu L, Wang Y, Liu X, et al. Tai chi exercise can ameliorate physical and mental health of patients with knee osteoarthritis: Systematic review and meta-analysis. Clin Rehabil 2021;35:64-79.  25. Hu YN, Chung YJ, Yu HK, Chen YC, Tsai CT, Hu GC. Effect of tai chi exercise on fall prevention in older adults: Systematic review and meta-analysis of randomized controlled trials. International Journal of Gerontology 2016;10:131-6.  26. Huang CY, Mayer PK, Wu MY, Liu DH, Wu PC, Yen HR. The effect of tai chi in elderly individuals with sarcopenia and frailty: A systematic review and meta-analysis of randomized controlled trials. Ageing Res Rev 2022;82:101747.  27. Huang Y, Liu X. Improvement of balance control ability and flexibility in the elderly tai chi chuan (tcc) practitioners: A systematic review and meta-analysis. Arch Gerontol Geriatr 2015;60:233-8.  28. Huang ZG, Feng YH, Li YH, Lv CS. Systematic review and meta-analysis: Tai chi for preventing falls in older adults. BMJ Open 2017;7:e013661.  29. Kong LJ, Lauche R, Klose P, et al. Tai chi for chronic pain conditions: A systematic review and meta-analysis of randomized controlled trials. Sci Rep 2016;6:25325.  30. Kuo CC, Wang CC, Chang WL, Liao TC, Chen PE, Tung TH. Clinical effects of baduanjin qigong exercise on cancer patients: A systematic review and meta-analysis on randomized controlled trials. Evid Based Complement Alternat Med 2021;2021:6651238.  31. Lai J, Cai Y, Yang L, Xia M, Cheng X, Chen Y. Effects of baduanjin exercise on motor function, balance and gait in parkinson's disease: A systematic review and meta-analysis. BMJ Open 2022;12.  32. Lauche R, Langhorst J, Dobos G, Cramer H. A systematic review and meta-analysis of tai chi for osteoarthritis of the knee. Complement Ther Med 2013;21:396-406.  33. Lee MS, Jun JH, Lim HJ, Lim HS. A systematic review and meta-analysis of tai chi for treating type 2 diabetes. Maturitas 2015;80:14-23.  34. Li GY, Wang W, Liu GL, Zhang Y. Effects of tai chi on balance and gait in stroke survivors: A systematic meta-analysis of randomized controlled trials. J Rehabil Med 2018;50:582-8.  35. Li H, Chen J, Xu G, et al. The effect of tai chi for improving sleep quality: A systematic review and meta-analysis. J Affect Disord 2020;274:1102-12.  36. Li Y, Zhang Y, Cui C, et al. The effect of tai chi exercise on motor function and sleep quality in patients with stroke: A meta-analysis. Int J Nurs Sci 2017;4:314-21.  37. Liang H, Luo S, Chen X, Lu Y, Liu Z, Wei L. Effects of tai chi exercise on cardiovascular disease risk factors and quality of life in adults with essential hypertension: A meta-analysis. Heart Lung 2020;49:353-63.  38. Lin H, Wan M, Ye Y, Zheng G. Effects of baduanjin exercise on the physical function of middle-aged and elderly people: A systematic review and meta-analysis of randomized controlled trials. BMC Complementary Medicine and Therapies 2023;23.  39. Lin R, Cui S, Yang J, et al. Effects of tai chi on patients with mild cognitive impairment: A systematic review and meta-analysis of randomized controlled trials. Biomed Res Int 2021;2021:5530149.  40. Liu F, Wang S. Effect of tai chi on bone mineral density in postmenopausal women: A systematic review and meta-analysis of randomized control trials. J Chin Med Assoc 2017;80:790-5.  41. Liu HH, Yeh NC, Wu YF, Yang YR, Wang RY, Cheng FY. Effects of tai chi exercise on reducing falls and improving balance performance in parkinson's disease: A meta-analysis. Parkinsons Dis 2019;2019:9626934.  42. Liu L, Tan H, Yu S, Yin H, Baxter GD. The effectiveness of tai chi in breast cancer patients: A systematic review and meta-analysis. Complement Ther Clin Pract 2020;38:101078.  43. Liu SJ, Ren Z, Wang L, Wei GX, Zou L. Mind⁻body (baduanjin) exercise prescription for chronic obstructive pulmonary disease: A systematic review with meta-analysis. Int J Environ Res Public Health 2018;15.  44. Liu X, Jiang C, Fan R, et al. The effect and safety of tai chi on bone health in postmenopausal women: A meta-analysis and trial sequential analysis. Front Aging Neurosci 2022;14:935326.  45. Liu YN, Wang L, Fan X, Liu S, Wu Q, Qian YL. A meta-analysis of the effects of tai chi on glucose and lipid metabolism in middle-aged and elderly diabetic patients: Evidence from randomized controlled trials. Evid Based Complement Alternat Med 2021;2021:6699935.  46. Liu Z, Hu H, Wen X, et al. Baduanjin improves neck pain and functional movement in middle-aged and elderly people: A systematic review and meta-analysis of randomized controlled trials. Front Med (Lausanne) 2022;9:920102.  47. Logghe IH, Verhagen AP, Rademaker AC, et al. The effects of tai chi on fall prevention, fear of falling and balance in older people: A meta-analysis. Prev Med 2010;51:222-7.  48. Luo XC, Liu J, Fu J, et al. Effect of tai chi chuan in breast cancer patients: A systematic review and meta-analysis. Front Oncol 2020;10:607.  49. Lyu D, Lyu X, Zhang Y, et al. Tai chi for stroke rehabilitation: A systematic review and meta-analysis of randomized controlled trials. Front Physiol 2018;9:983.  50. Ni X, Chan RJ, Yates P, Hu W, Huang X, Lou Y. The effects of tai chi on quality of life of cancer survivors: A systematic review and meta-analysis. Support Care Cancer 2019;27:3701-16.  51. Ni X, Liu S, Lu F, Shi X, Guo X. Efficacy and safety of tai chi for parkinson's disease: A systematic review and meta-analysis of randomized controlled trials. PLoS One 2014;9:e99377.  52. Pan L, Yan J, Guo Y, Yan J. Effects of tai chi training on exercise capacity and quality of life in patients with chronic heart failure: A meta-analysis. Eur J Heart Fail 2013;15:316-23.  53. Pan X, Tian L, Yang F, et al. Tai chi as a therapy of traditional chinese medicine on reducing blood pressure: A systematic review of randomized controlled trials. Evid Based Complement Alternat Med 2021;2021:4094325.  54. Pan XH, Mahemuti A, Zhang XH, et al. Effect of tai chi exercise on blood lipid profiles: A meta-analysis of randomized controlled trials. J Zhejiang Univ Sci B 2016;17:640-8.  55. Pan Y, Yang K, Shi X, Liang H, Zhang F, Lv Q. Tai chi chuan exercise for patients with breast cancer: A systematic review and meta-analysis. Evid Based Complement Alternat Med 2015;2015:535237.  56. Qin J, Zhang Y, Wu L, et al. Effect of tai chi alone or as additional therapy on low back pain: Systematic review and meta-analysis of randomized controlled trials. Medicine (Baltimore) 2019;98:e17099.  57. Ren X, Li Y, Yang X, et al. The effects of tai chi training in patients with heart failure: A systematic review and meta-analysis. Front Physiol 2017;8:989.  58. Sani NA, Yusoff SSM, Norhayati MN, Zainudin AM. Tai chi exercise for mental and physical well-being in patients with depressive symptoms: A systematic review and meta-analysis. Int J Environ Res Public Health 2023;20.  59. Shao BY, Zhang XT, Vernooij RWM, et al. The effectiveness of baduanjin exercise for hypertension: A systematic review and meta-analysis of randomized controlled trials. BMC Complementary Medicine and Therapies 2020;20.  60. Shu C, Feng S, Cui Q, Cheng S, Wang Y. Impact of tai chi on crp, tnf-alpha and il-6 in inflammation: A systematic review and meta-analysis. Ann Palliat Med 2021;10:7468-78.  61. Si Y, Wang C, Yin H, et al. Tai chi chuan for subjective sleep quality: A systematic review and meta-analysis of randomized controlled trials. Evid Based Complement Alternat Med 2020;2020:4710527.  62. Song S, Yu J, Ruan Y, Liu X, Xiu L, Yue X. Ameliorative effects of tai chi on cancer-related fatigue: A meta-analysis of randomized controlled trials. Support Care Cancer 2018;26:2091-102.  63. Sun C, Qi B, Huang X, et al. Baduanjin exercise: A potential promising therapy toward osteoporosis. Front Med (Lausanne) 2022;9:935961.  64. Sun Z, Chen H, Berger MR, Zhang L, Guo H, Huang Y. Effects of tai chi exercise on bone health in perimenopausal and postmenopausal women: A systematic review and meta-analysis. Osteoporos Int 2016;27:2901-11.  65. Tan T, Meng Y, Lyu JL, et al. A systematic review and meta-analysis of tai chi training in cardiorespiratory fitness of elderly people. Evid Based Complement Alternat Med 2022;2022:4041612.  66. 魏洪悦, 靳英辉, 谷晓玲, et al. 太极拳对心力衰竭患者作用效果的系统评价. 中国循证医学杂志 2017;17:677-84.  67. Wang D, Wang P, Lan K, Zhang Y, Pan Y. Effectiveness of tai chi exercise on overall quality of life and its physical and psychological components among older adults: A systematic review and meta-analysis. Braz J Med Biol Res 2020;53:e10196.  68. Wang D, Xu J. Updated meta-analysis assessing effects of baduanjin on cardiopulmonary functions of patients with coronary heart disease. Evidence-based Complementary and Alternative Medicine 2022;2022.  69. Wang K, Liu S, Kong Z, Zhang Y, Liu J. Mind-body exercise (wuqinxi) for patients with chronic obstructive pulmonary disease: A systematic review and meta-analysis of randomized controlled trials. Int J Environ Res Public Health 2018;16.  70. Wang LC, Ye MZ, Xiong J, Wang XQ, Wu JW, Zheng GH. Optimal exercise parameters of tai chi for balance performance in older adults: A meta-analysis. J Am Geriatr Soc 2021;69:2000-10.  71. Wang Y, Yan J, Zhang P, Yang P, Zhang W, Lu M. Tai chi program to improve glucose control and quality of life for the elderly with type 2 diabetes: A meta-analysis. Inquiry 2022;59:469580211067934.  72. Wehner C, Blank C, Arvandi M, Wehner C, Schobersberger W. Effect of tai chi on muscle strength, physical endurance, postural balance and flexibility: A systematic review and meta-analysis. BMJ Open Sport Exerc Med 2021;7:e000817.  73. Wen J, Lin T, Cai Y, et al. Baduanjin exercise for type 2 diabetes mellitus: A systematic review and meta-analysis of randomized controlled trials. Evidence-based Complementary and Alternative Medicine 2017;2017.  74. Wu B, Ding Y, Zhong B, Jin X, Cao Y, Xu D. Intervention treatment for myocardial infarction with tai chi: A systematic review and meta-analysis. Arch Phys Med Rehabil 2020;101:2206-18.  75. Wu S, Chen J, Wang S, Jiang M, Wang X, Wen Y. Effect of tai chi exercise on balance function of stroke patients: A meta-analysis. Med Sci Monit Basic Res 2018;24:210-5.  76. Wu W, Liu X, Wang L, Wang Z, Hu J, Yan J. Effects of tai chi on exercise capacity and health-related quality of life in patients with chronic obstructive pulmonary disease: A systematic review and meta-analysis. Int J Chron Obstruct Pulmon Dis 2014;9:1253-63.  77. Xia TW, Yang Y, Li WH, Tang ZH, Li ZR, Qiao LJ. Different training durations and styles of tai chi for glucose control in patients with type 2 diabetes: A systematic review and meta-analysis of controlled trials. BMC Complement Altern Med 2019;19:63.  78. Xiang Y, Lu L, Chen X, Wen Z. Does tai chi relieve fatigue? A systematic review and meta-analysis of randomized controlled trials. PLoS One 2017;12:e0174872.  79. Xiao L, Duan H, Li P, Wu W, Shan C, Liu X. A systematic review and meta-analysis of liuzijue in stable patients with chronic obstructive pulmonary disease. BMC Complement Med Ther 2020;20:308.  80. Xiong X, Wang P, Li S, Zhang Y, Li X. Effect of baduanjin exercise for hypertension: A systematic review and meta-analysis of randomized controlled trials. Maturitas 2015;80:370-8.  81. Xu S, Zhang D, He Q, et al. Efficacy of liuzijue qigong in patients with chronic obstructive pulmonary disease: A systematic review and meta-analysis. Complement Ther Med 2022;65:102809.  82. Yan JH, Gu WJ, Sun J, Zhang WX, Li BW, Pan L. Efficacy of tai chi on pain, stiffness and function in patients with osteoarthritis: A meta-analysis. PLoS One 2013;8:e61672.  83. Yan JH, Guo YZ, Yao HM, Pan L. Effects of tai chi in patients with chronic obstructive pulmonary disease: Preliminary evidence. PLoS One 2013;8:e61806.  84. Yang G, Li W, Klupp N, et al. Does tai chi improve psychological well-being and quality of life in patients with cardiovascular disease and/or cardiovascular risk factors? A systematic review. BMC Complement Med Ther 2022;22:3.  85. Yang J, Zhang L, Tang Q, et al. Tai chi is effective in delaying cognitive decline in older adults with mild cognitive impairment: Evidence from a systematic review and meta-analysis. Evid Based Complement Alternat Med 2020;2020:3620534.  86. Yang M, Yang J, Gong M, Luo R, Lin Q, Wang B. Effects of tai chi on sleep quality as well as depression and anxiety in insomnia patients: A meta-analysis of randomized controlled trials. Int J Environ Res Public Health 2023;20.  87. Yang WY, Xu Y, Ye L, et al. Effects of baduanjin exercise on quality-of-life and exercise capacity in patients with heart failure: A systematic review and meta-analysis. Complementary therapies in clinical practice 2023;50:101675.  88. Yang Y, Li JH, Xu NJ, Yang WY, Liu J. Meta-analysis of elderly lower body strength: Different effects of tai chi exercise on the knee joint-related muscle groups. Evid Based Complement Alternat Med 2021;2021:8628182.  89. Ye XX, Ren ZY, Vafaei S, et al. Effectiveness of baduanjin exercise on quality of life and psychological health in postoperative patients with breast cancer: A systematic review and meta-analysis. Integr Cancer Ther 2022;21:15347354221104092.  90. Yin Y, Yu Z, Wang J, Sun J. Effects of the different tai chi exercise cycles on patients with essential hypertension: A systematic review and meta-analysis. Front Cardiovasc Med 2023;10:1016629.  91. You Y, Liu J, Tang M, Wang D, Ma X. Effects of tai chi exercise on improving walking function and posture control in elderly patients with knee osteoarthritis: A systematic review and meta-analysis. Medicine (Baltimore) 2021;100:e25655.  92. Yu L, Liu F, Nie P, Shen C, Chen J, Yao L. Systematic review and meta-analysis of randomized controlled trials assessing the impact of baduanjin exercise on cognition and memory in patients with mild cognitive impairment. Clin Rehabil 2021;35:492-505.  93. Yu X, Wu X, Hou G, Han P, Jiang L, Guo Q. The impact of tai chi on motor function, balance, and quality of life in parkinson's disease: A systematic review and meta-analysis. Evid Based Complement Alternat Med 2021;2021:6637612.  94. Zeng ZP, Liu YB, Fang J, Liu Y, Luo J, Yang M. Effects of baduanjin exercise for knee osteoarthritis: A systematic review and meta-analysis. Complement Ther Med 2020;48:102279.  95. Zhang F, Zhao J, Jiang N, Zhai Q, Hu J, Zhang J. Meta-analysis of tai chi chuan in treating lumbar spondylosis and back pain. Appl Bionics Biomech 2022;2022:2759977.  96. Zhang Y, Chai Y, Pan X, Shen H, Wei X, Xie Y. Tai chi for treating osteopenia and primary osteoporosis: A meta-analysis and trial sequential analysis. Clin Interv Aging 2019;14:91-104.  97. Zhang Y, Lu S. Effects of traditional chinese exercises on mental health in individuals with drug rehabilitee: A systematic review and meta-analysis. Front Public Health 2022;10:944636.  98. Zheng W, Li Q, Lin J, et al. Tai chi for schizophrenia: A systematic review. Shanghai Arch Psychiatry 2016;28:185-94.  99. Zheng X, Wu X, Liu Z, et al. The influences of tai chi on balance function and exercise capacity among stroke patients: A meta-analysis. Evid Based Complement Alternat Med 2021;2021:6636847.  100. Zhong D, Li J, Yang H, et al. Tai chi for essential hypertension: A systematic review of randomized controlled trials. Curr Hypertens Rep 2020;22:25.  101. Zhou Y, Zhao ZH, Fan XH, Li WH, Chen Z. Different training durations and frequencies of tai chi for bone mineral density improvement: A systematic review and meta-analysis. Evid Based Complement Alternat Med 2021;2021:6665642.  102. Zhou Z, Zhou R, Li K, et al. Effects of tai chi on physiology, balance and quality of life in patients with type 2 diabetes: A systematic review and meta-analysis. J Rehabil Med 2019;51:405-17.  103. Zhu PA, Lu QQ, Li ZL, et al. Efficacy of tai chi on lower limb function of parkinson's disease patients: A systematic review and meta-analysis. Front Aging Neurosci 2023;15:1096417.  104. Zou L, SasaKi JE, Wang H, Xiao Z, Fang Q, Zhang M. A systematic review and meta-analysis baduanjin qigong for health benefits: Randomized controlled trials. Evid Based Complement Alternat Med 2017;2017:4548706.  105. Zou L, Wang C, Chen K, et al. The effect of taichi practice on attenuating bone mineral density loss: A systematic review and meta-analysis of randomized controlled trials. Int J Environ Res Public Health 2017;14.  106. Zou L, Zhang Y, Sasaki JE, et al. Wuqinxi qigong as an alternative exercise for improving risk factors associated with metabolic syndrome: A meta-analysis of randomized controlled trials. Int J Environ Res Public Health 2019;16.  107. 曹青青, 杨丽丽, 李文林, 等. 易筋经防治老年人原发性骨质疏松的meta分析. 世界中医药 2018;13:2647-52.  108. 曾令烽, 杨伟毅, 梁桂洪,等. 传统太极功法干预对改善骨密度流失疗效及安全性的系统评价. 中国组织工程研究 2019;23:4420-8.  109. 陈金辉. 八段锦对大学生心理健康干预效果的系统评价. 龙岩学院学报 2021;39:102-11+17.  110. 陈锦秀，李晓军，陈婷玉，等. 太极拳对老年人单足站立时间影响差异性的meta分析. 康复学报 2016;26.  111. 陈珊杉，尹甜甜，邓士琳，等. 太极拳运动对2型糖尿病患者身体机能影响研究的meta分析. 体育科技文献通报 2022;30.  112. 陈笑银, 赵经营, 张广清, 等. 八段锦对高血压患者血压影响的meta分析. 中国医药导报 2018;15:137-40.  113. 陈心铭, 聂平英, 陈苏莲, 等. 八段锦改善mci患者注意力临床随机对照试验的系统评价与meta分析. 按摩与康复医学 2022;13:55-61.  114. 陈燕华, 肖璐, 赵容, 等. 八段锦对稳定期慢性阻塞性肺疾病患者康复效果影响的meta分析. 中国康复医学杂志 2018;33:451-6.  115. 陈艺曦, 云洁, 刘芯言, 等. 八段锦防治绝经后女性骨质疏松的meta分析. 按摩与康复医学 2022;13:41-7.  116. 陈祖森, 郑丽维, 杨晨晨, 等. 八段锦运动对高血压患者干预效果的meta分析. 解放军护理杂志 2018;35:1-8.  117. 戴文康, 谢瑞, 常晓娟, 等. 八段锦治疗神经根型颈椎病的系统评价与meta分析. 天津中医药 2020;37:422-8.  118. 邓叶龙, 孔令俊, 刘朝晖, 等. 太极拳锻炼防治骨质疏松症的meta分析. 中医正骨 2021;33:44-50+3.  119. 都文渊, 赵玉斌, 姚建景, 等. 八段锦对老年人平衡功能影响的meta分析. 临床医学研究与实践 2020;5:1-4.  120. 范铜钢，李文龙，向秋平，等. 太极拳对轻度认知功能障碍患者认知功能影响的meta分析. 中医临床研究 2021;13.  121. 方磊，李振瑞，占超，等. 预防老年人跌倒的最佳太极拳运动量的meta分析. 时珍国医国药 2021;32.  122. 高亮，王莉华. 太极拳锻炼对老年人copd患者干预效果的meta分析. 广州体育学院学报 2020;40.  123. 葛乐，董晋，纪云哲，等. 太极拳对稳定期中老年人慢性阻塞性肺疾病患者康复疗效的meta分析. 吉林体育学院学报 2021;37.  124. 郭佳颖, 林少鸿, 郭佳瑶, 等. 八段锦对轻度认知障碍患者记忆力影响的系统评价与meta分析. 中国疗养医学 2022;31:8-13.  125. 韩梅, 赖永明月, 苏雨晨, 王飞. 六字诀功法对中老年慢性阻塞性肺疾病稳定期患者肺功能康复效果的meta分析. 中医临床研究:1-6.  126. 韩燕, 王清馨, 罗丹, 薛俊林. 八段锦对稳定期慢性阻塞性肺疾病患者肺康复影响meta分析. 护理学报 2017;24:15-9.  127. 何明星, 钟冬灵, 刘栖岑, 等. 太极拳训练对帕金森患者平衡功能影响的meta分析. 世界最新医学信息文摘 2019;19:6-10.  128. 侯江涛, 郑鸿铭, 严梓萁, 等. 八段锦干预失眠症患者疗效的meta分析. 广州体育学院学报 2022;42:59-69.  129. 侯美金 李翔，王芗斌，等. 太极拳治疗下腰痛临床疗效的meta分析. 康复学报 2017;27.  130. 胡大一，李建超，吴燕婷，等. 太极拳对心力衰竭患者心功能及生活质量影响的meta分析. 慢性病学杂志 2018;0.  131. 姜军，苏中军. 太极拳锻炼对肌力改善效果的meta分析. 中国康复医学杂志 2020;35.  132. 金昌德，李玉峰，张亚静，等. 太极拳运动疗法改善心肌梗死患者心功能的meta分析. 中国循证心血管医学杂志 2020;12.  133. 金昌德 李A张A崔A刘A雷A刘A孟A. 太极运动对脑卒中患者运动功能及睡眠质量影响的系统评价. 国际护理科学（英文） 2017.  134. 金成吉, 张自云, 解超. 太极拳对中老年原发性高血压患者血压水平影响的meta分析. 现代预防医学 2018;45:3446-51.  135. 金奕 张琪，宋慧敏，等. 太极拳对轻度认知障碍老年人认知功能干预效果的meta分析. 中国护理管理 2020;20.  136. 李海鸿，黎燕，任军丽，等. 八段锦治疗颈椎病颈痛效果的meta分析. 中文科技期刊数据库(引文版)医药卫生 2022.  137. 李恩耀，于谦，李立国，等. 太极拳训练改善在校大学生抑郁情绪的meta分析. 国际精神病学杂志 2019;0.  138. 李黔峰, 李少洪, 张伟, 李振帆, 黎洁莹, 刘同刚. 八段锦锻炼对慢性心力衰竭患者疗效的meta分析. 岭南急诊医学杂志 2022;27:557-61.  139. 李小燕, 云洁, 何杰, 等. 八段锦对骨质疏松症患者干预效果的meta分析. 中国骨质疏松杂志 2020;26:37-43+84.  140. 李晓，蔡璐. 太极拳治疗原发性高血压疗效meta分析. 中医药临床杂志 2016;0.  141. 刘海, 沈娅妮, 刘晓梅, 等. 五禽戏对稳定期慢性阻塞性肺疾病患者有效性的meta分析. 西部中医药 2023;36:53-8.  142. 刘景新 王继红，刘晓丹，等. 太极拳对老年慢性阻塞性肺疾病患者肺功能和运动耐力影响的meta分析. 中国组织工程研究 2015;19.  143. 刘军 谢辉，潘建科，等. 太极拳治疗骨关节炎的系统评价和meta分析. 中华中医药杂志 2016;31.  144. 刘文君, 郝建英. 太极运动治疗骨关节患者关节疼痛、僵硬及功能障碍的系统评价及meta分析. 中国老年学杂志 2020;40:3471-5.  145. 刘雅莉 赵媛，王燕. 太极拳运动对老年人平衡功能和跌倒预防效果的meta分析. 中国循证医学杂志 2013;13.  146. 刘跃, 蒋运兰, 林玉珠, 等. 八段锦治疗颈型颈椎病有效性的meta分析. 湖南中医杂志 2021;37:152-5.  147. 卢峰, 王世聪. “六字诀”呼吸操对慢性阻塞性肺疾病稳定期患者康复效果的meta分析. 中医研究 2021;34:45-50.  148. 罗乃搏. 八段锦对冠心病患者生活质量影响的meta分析. 辽宁中医药大学; 2020.  149. 罗乃搏, 董波. 八段锦对冠心病病人焦虑、抑郁及心绞痛发作频率影响的meta分析. 中西医结合心脑血管病杂志 2021;19:2133-7.  150. 马沐佳，李欣欣，刘卉. 太极拳与其他运动降低老年人跌倒风险的meta分析. 中国康复理论与实践 2022;28.  151. 马占科, 曹宇璐. 八段锦在糖尿病周围神经病变中应用效果的meta分析. 宁夏师范学院学报 2022;43:51-9.  152. 邱定荣, 林小丽, 赵经营, 陈笑银. 八段锦改善抑郁症状meta分析. 新中医 2019;51:51-4.  153. 石丽红，黄依杰，金荣疆，等. 太极拳对中老年人跌倒及平衡功能影响的meta分析. 中国循证医学杂志 2020;20.  154. 宋久存 陈浩生. 太极拳运动对中轻度帕金森患者平衡功能影响的meta分析. 体育科技文献通报 2022;30.  155. 宋咪, 徐月, 宋杰, 等. 太极对老年人跌倒预防效果的meta分析. 海南医学院学报 2020;26:545-51.  156. 孙君志，尹贻锟，尹逊伟，等. 太极拳锻炼周期对原发性高血压的血压及心血管危险因素的影响:系统性评价和meta分析. 临床荟萃 2022;37.  157. 覃林, 韦霞, 刘琳, 等. 太极运动对脑卒中患者运动、情绪及生活质量影响的系统评价和meta分析. 中国组织工程研究 2016;20:297-303.  158. 唐青, 郭瑜洁, 李萍, 等. 太极拳在2型糖尿病患者中应用效果的meta分析. 现代预防医学 2017;44:2516-21.  159. 陶醉 虞芬，钟清玲，等. 太极拳运动对轻中度帕金森病患者影响的meta分析. 中国康复医学杂志 2018;33.  160. 田素斋 张海娇，孙卫格，等. 太极拳干预非特异性腰背痛的meta分析. 中国民族民间医药 2021;30.  161. 王安铸, 马晓昌. 八段锦干预心力衰竭患者疗效的meta分析. 世界中医药 2020;15:1939-45+52.  162. 王岑依, 焦欣荣, 任园园, 等. 太极运动对健康老年人群运动功能影响的meta分析. 体育科研 2022;43:88-97.  163. 王建月, 丁勇, 李云卿. 八段锦对中老年人生理机能影响的meta分析. 吉首大学学报(自然科学版) 2016;37:73-7+82.  164. 王梅杰, 廖春满, 张正媚, 等. 八段锦对2型糖尿病患者血糖血脂水平等辅助治疗效果影响的meta分析. 北京中医药 2021;40:179-84.  165. 王齐. 太极拳治疗2型糖尿病随机对照实验的系统评价与meta分析. 北京体育大学; 2017.  166. 王尚全，梁龙，韩涛，等. 太极拳锻炼防治骨质疏松症效果的meta 分析. 中国骨质疏松杂志 2019;25.  167. 邹宇, 王芳, 巩文花, 等. 八段锦对2型糖尿病患者焦虑抑郁情绪及血糖干预效果的meta分析. 湖南中医杂志 2019;35:113-7.  168. 朱胜伶, 王传池, 何嘉莉, 等. 八段锦对糖尿病患者糖脂代谢干预效果的meta分析. 世界科学技术-中医药现代化 2020;22:1478-86.  169. 郑清香, 葛莉, 谭景予, 等. 八段锦对脑卒中患者肢体功能康复影响的meta分析. 解放军护理杂志 2017;34:1-7.  170. 赵怡茜, 吴敏. 八段锦干预稳定性心绞痛的meta分析及grade评价. 联勤军事医学 2023;37:248-55.  171. 张颖 王晨，杨坚，等. 短期的太极拳结合常规康复训练治疗脑卒中偏瘫患者平衡能力、运动功能的meta分析. 中国康复医学杂志 2018;33.  172. 张亚青, 张康, 谢凯, 等. 六字诀康复法对稳定期慢性阻塞性肺疾病患者的疗效及安全性的系统评价及meta分析. 中医临床研究 2021;13:126-31.  173. 张竣玮, 方朝晖, 汪四海, 等. 八段锦对2型糖尿病临床疗效meta分析. 中医药临床杂志 2021;33:83-6.  174. 张建国, 唐纯志, 孔令朔. 太极拳运动对老年人认知功能影响的系统评价与meta分析. 中医杂志 2017;58:1473-7.  175. 张宝珍, 王秋岩, 董林森, 等. 中国健身气功八段锦对中老年人血脂影响的meta分析. 西南军医 2019;21:241-6.  176. 袁雷, 云洁, 刘芯言, 等. 五禽戏对慢性阻塞性肺疾病病人肺功能干预效果的meta分析. 循证护理 2021;7:1869-74.  177. 袁冰华，龙换平. 太极拳对癌因性疲乏干预效果的meta分析. 中国疗养医学 2022;31.  178. 尹剑春，岳彩超，陈翔. 太极拳锻炼对个体疲劳的干预效果——基于随机对照试验的系统评价与meta分析. 湖北体育科技 2021;40.  179. 叶学球 张明军. 太极拳练习对中老年人血压影响的meta分析. 福建体育科技 2015;34.  180. 杨晓丽, 蔡益民, 吴贤琳, 等. 八段锦对冠心病患者心脏康复干预效果的meta分析. 医学信息 2019;32:70-4.  181. 杨继鹏, 刘璟莹, 吕纹良, 等. 健身气功八段锦治疗2型糖尿病疗效的meta分析. 中华中医药杂志 2015;30:1307-9.  182. 许美丽，刘晓慧，王申，等. 六字诀对慢性阻塞性肺疾病稳定期患者影响的meta分析. 中国实用护理杂志 2015;31.  183. 薛雅卓，葛瑶，秦超，等. 太极拳对t2dm患者血糖及脂蛋白代谢水平影响的meta分析. 泰山医学院学报 2020;41.  184. 许丽璇, 刘建璟, 徐莹银, 等. 八段锦运动对维持性血液透析患者睡眠质量改善有效性的meta分析. 基层中医药 2022;1:57-63.  185. 徐长力, 吕浩源, 张涛, 等. 健身气功五禽戏治疗老年性骨质疏松症meta分析. 亚太传统医药 2019;15:164-7.  186. 徐文君, 涂惠, 熊晓云, 等. 八段锦对冠心病患者心脏康复功效的meta分析. 临床护理杂志 2021;20:2-6.  187. 徐聆, 尹雨晴, 陈燕. 八段锦干预冠心病pci术后患者疗效的meta分析. 中医药导报 2020;26:150-5+74.  188. 邢华, 沈佳韵, 邵盛, 等. 六字诀对于肺功能康复影响的meta分析. 北京中医药 2021;40:1405-9.  189. 谢秋蓉, 夏锐, 梁正侠, 李雪梅, 吴劲松, 吴成晖. 八段锦对慢性阻塞性肺部疾病干预效果的系统评价与meta分析. 福建中医药 2020;51:80-8.  190. 吴玉龙, 安军明, 李彦娇, 孙慧, 殷之珺, 杨敏. 八段锦运动疗法治疗颈椎病临床疗效的meta分析. 湖南中医杂志 2022;38:128-33.  191. 吴千言, 章莹. 中医传统功法八段锦干预治疗失眠的系统评价和meta分析. 中医临床研究 2022;14:127-30.  192. 谢榆, 魏刚, 郭云柯, 等. 太极治疗膝骨关节炎系统评价及meta分析. 中国康复医学杂志 2015;30:483-9.  193. 魏璐璐, 朱泓吉, 朱琳, 刘景新. 八段锦对2型糖尿病患者血糖影响的meta分析. 湘南学院学报 2021;42:71-5+95.  194. 王瑶, 郭彬, 孙露, 等. 八段锦对慢性心力衰竭患者生活质量影响的meta分析. 按摩与康复医学 2019;10:45-9.  195. 王雪冰, 冯连世. 健身气功五禽戏对成年人血脂影响的meta分析. 中国运动医学杂志 2017;36:156-63+82. |
| --- |
