# Supplementary material for: Evidence map of traditional Chinese exercises
Source: Front Public Health. 2024 Sep 18;12:1347201. doi: 10.3389/fpubh.2024.1347201 (PMC11445016; doi:10.3389/fpubh.2024.1347201)
Supplement: Supplementary file 8 [file Table_5.docx]

**Supplementary Table 5 Evidence map**

| **3.6.1 Musculoskeletal system and connective tissue disorders (included 39 SRs)** | **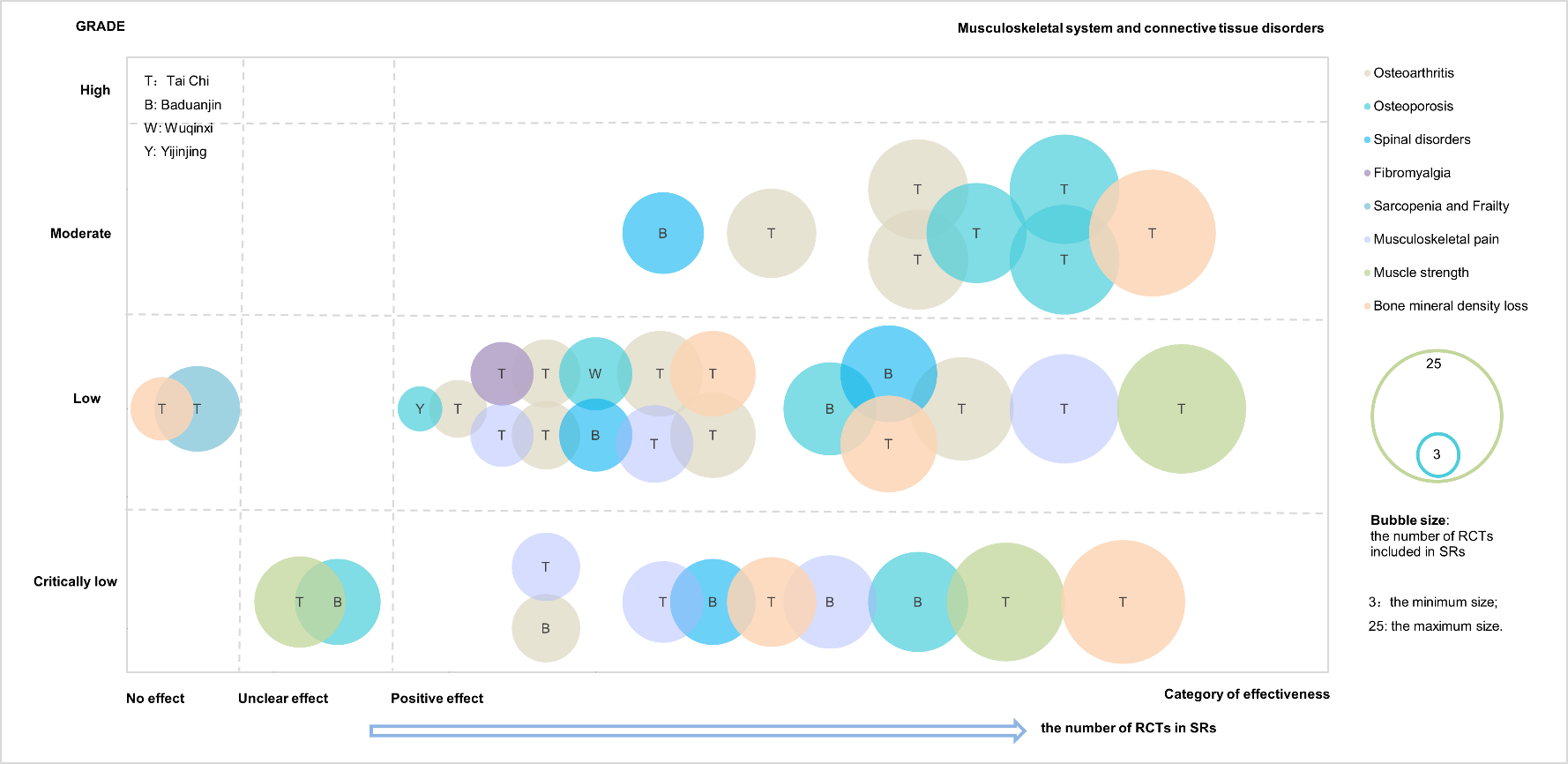**  **Supplementary Figure 4.** The evidence map of traditional Chinese exercises for musculoskeletal system and connective tissue disorders.  Note: The size of bubbles is determined by the number of RCTs included in SRs. The color of bubbles is represented different diseases and conditions. The level of GRADE: critically low, low, moderate, high. No effect: no statistical difference between traditional Chinese exercises and controls. Unclear effect: results showed mixed findings. Positive effect: effect estimates of traditional Chinese exercises are significantly positive. The bubble labels of T, B, W, L and Y represent Tai Chi, Baduanjin, Wuqinxi, Liuzijue and Yijinjing. | |
| --- | --- | --- |
|  | **General summary** | **Effect** |
|  | **① Interventions:**  Tai Chi, Baduanjin, Wuqinxi, and Yijinjing;  **② Diseases/symptoms:**  Osteoarthritis, osteoporosis, bone mineral density loss, musculoskeletal pain, Spinal disorders, muscle strength, fibromyalgia and sarcopenia;  **③ Effects and evidence quality:**  89.7% of the SRs indicated clinical benefits of traditional Chinese exercises in these diseases/symptoms, and the quality of evidence was critically low to moderate. | **1. Positive effect:**  **1.1 Tai Chi:**  **1.1.1 Osteoporosis:** bone mineral density (moderate quality evidence);  **1.1.2 Osteoarthritis:** WOMAC scale includes the pain, stiffness and function domains (low to moderate quality evidence);  **1.1.3 Musculoskeletal pain** (critically low to low quality evidence);  **1.1.4 Fibromyalgia:** fibromyalgia impact questionnaire (FIQ): used to assess the health status of patients with fibromyalgia (low quality evidence).  **1.2 Baduanjin:**  **1.2.1 Spinal disorders:** overall response rate (critically low to moderate quality);  **1.2.2 Musculoskeletal pain** (critically low quality evidence).  **1.3 Wuqinxi and Yijinjing:**  **1.3.1 osteoarthritis** (low quality evidence). |
|  |  | **2. Unclear effect:**  **2.1 Tai Chi: Muscle strength; Bone mineral density loss.**  **2.2 Baduanjin: Osteoporosis.** |
|  |  | **3. No effect:**  **3.1 Tai Chi: Sarcopenia**: muscle mass and other outcomes (low quality evidence) |
| **3.6.2 Circulatory system diseases**  **(included 33 SRs)** | **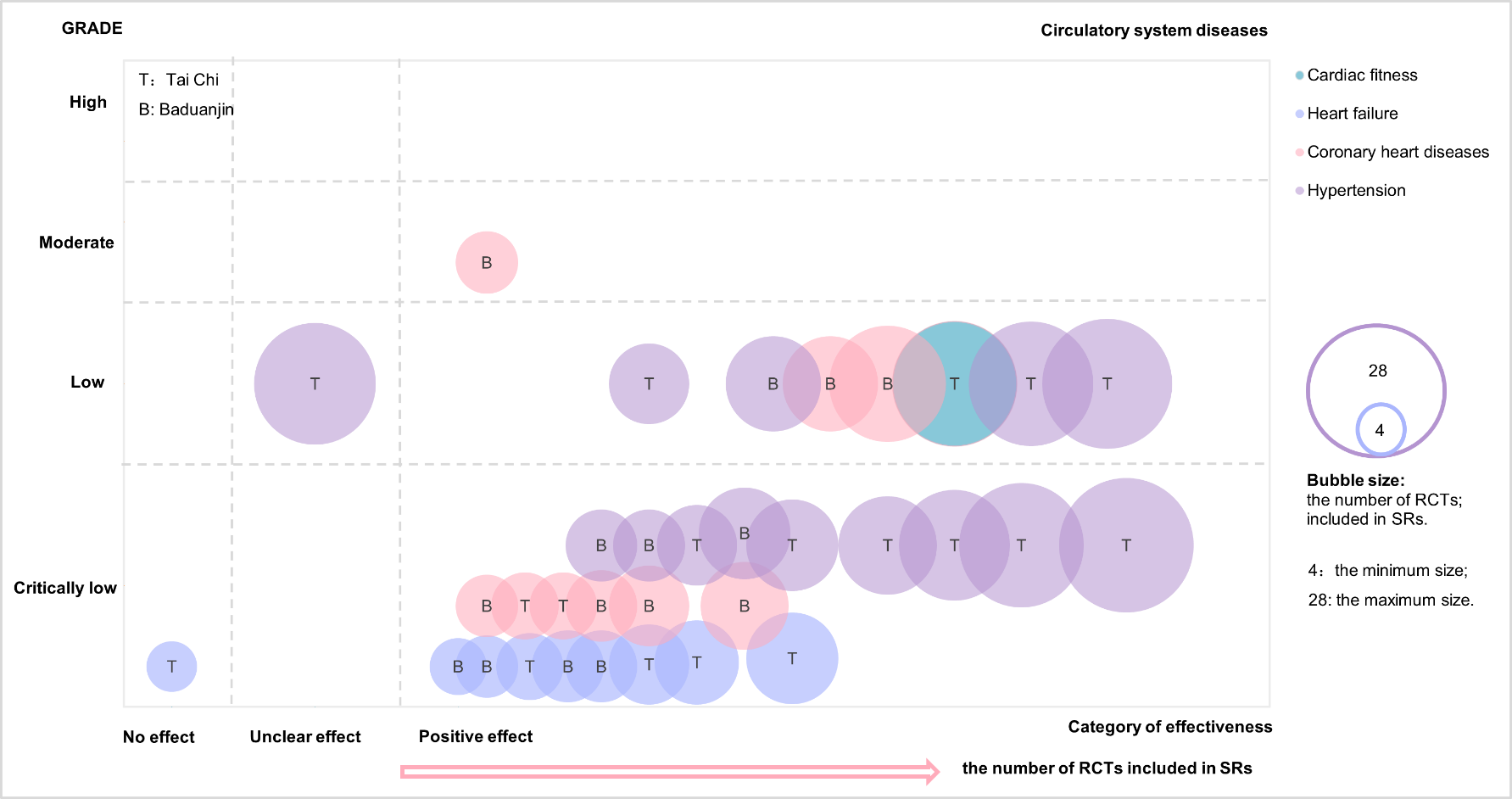**  **Supplementary Figure 5.** The evidence map of traditional Chinese exercises for circulatory system diseases.  Note: The size of bubbles is determined by the number of RCTs included in SRs. The color of bubbles is represented different diseases and conditions. The level of GRADE: critically low, low, moderate, high. No effect: no statistical difference between traditional Chinese exercises and controls. Unclear effect: results showed mixed findings. Positive effect: effect estimates of traditional Chinese exercises are significantly positive. The bubble labels of T, B, W, L and Y represent Tai Chi, Baduanjin, Wuqinxi, Liuzijue and Yijinjing. | |
|  | **General summary** | **Effect** |
|  | **① Interventions:**  Tai Chi, Baduanjin;  **② Diseases/symptoms:**  Hypertension, heart failure, coronary heart diseases, and cardiac fitness;  **③ Effects and evidence quality:**  93.9% of SRs suggested that traditional Chinese exercises had clinical benefits in these diseases/symptoms with critically low to moderate quality evidence. | **1. Positive effect:**  **1.1 Tai Chi:**  **1.1.1 Cardiac functions:** heart rate and other functions of elderly;  **1.1.2 Coronary heart diseases:** the 6-Minute walking test (6MWD) which reflects cardiopulmonary function.  **1.2 Baduanjin:**  **1.2.1 Angina pectoris:** reduce the frequency of angina pectoris;  **1.2.2 Coronary heart diseases:** promote cardiopulmonary health;  **1.2.3 Hypertension:** SBP and DBP;  **1.2.4 Heart failure:** cardiopulmonary health and quality of life. |
|  |  | **2. Unclear effect:**  **2.1 Tai Chi: Hypertension; Heart failure** |
| **3.6.3 Endocrine, nutritional, or metabolic diseases**  **(included 26 SRs)** | **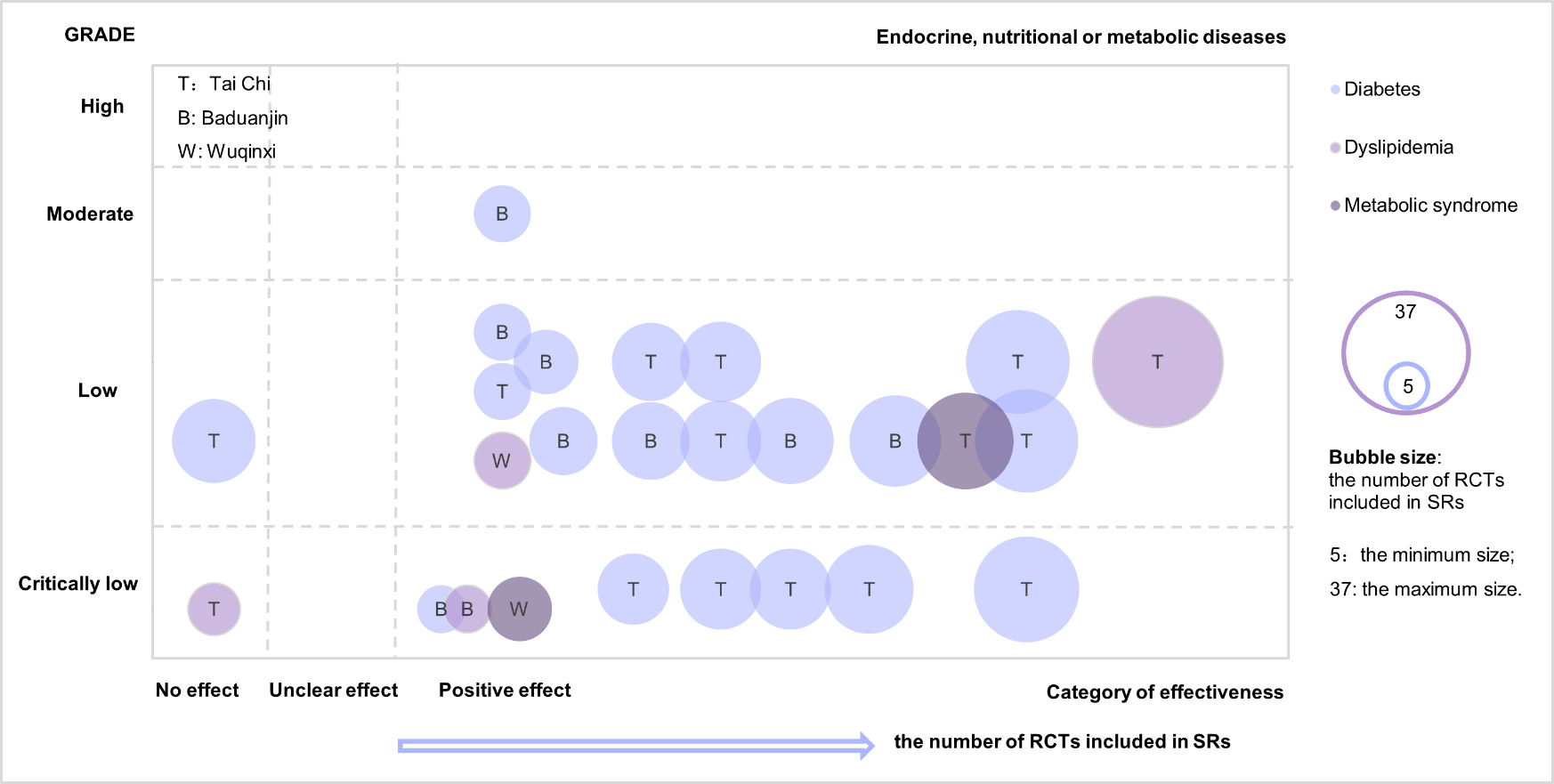**  **Supplementary Figure 6.** The evidence map of traditional Chinese exercises for endocrine, nutritional or metabolic diseases.  Note: The size of bubbles is determined by the number of RCTs included in SRs. The color of bubbles is represented different diseases and conditions. The level of GRADE: critically low, low, moderate, high. No effect: no statistical difference between traditional Chinese exercises and controls. Unclear effect: results showed mixed findings. Positive effect: effect estimates of traditional Chinese exercises are significantly positive. The bubble labels of T, B, W, L and Y represent Tai Chi, Baduanjin, Wuqinxi, Liuzijue and Yijinjing. | |
|  | **General summary** | **Effect** |
|  | **① Interventions:**  Tai Chi, Baduanjin and Wuqinxi;  **② Diseases/symptoms:**  Metabolic diseases of diabetes, hyperlipidemia, and metabolic syndrome;  **③ Effects and evidence quality:**  92.3% of the SRs demonstrated clinical benefits of traditional Chinese exercises for these diseases with critically low to moderate quality evidence. | **1. Positive effect:**  **1.1 Tai Chi:**  **1.1.2 Metabolic syndrome:** blood pressure, lipid and glucose outcomes;  **1.2 Baduanjin:**  **1.2.1 Diabetes:** fasting blood glucose (FBG) and glycosylated hemoglobin (HbA1c);  **1.2.2 Hyperlipidemia:** TC, TG, HDL-C and LDL-C;  **1.3 Wuqinxi:**  **1.3.1 hyperlipidemia:** lipid outcomes;  **1.3.2 Metabolic syndrome:** blood pressure, lipid and glucose outcomes; |
|  |  | **2. Unclear effect:**  **2.1 Tai Chi:** diabetes and hyperlipidemia |
| **3.6.4 Respiratory system diseases**  **(included 23 SRs)** | **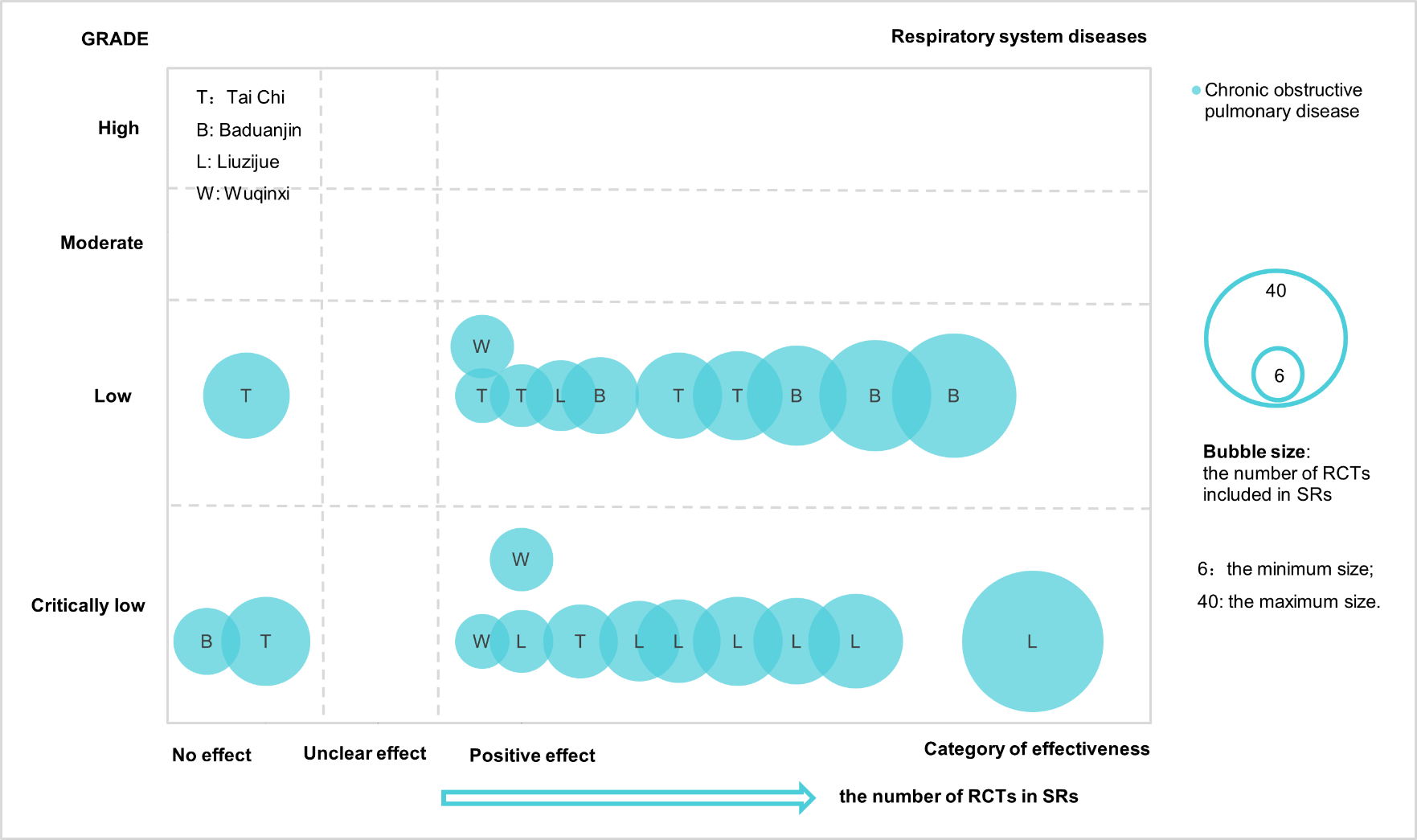**  **Supplementary Figure 7.** The evidence map of traditional Chinese exercises for respiratory system diseases.  Note: The size of bubbles is determined by the number of RCTs included in SRs. The color of bubbles is represented different diseases and conditions. The level of GRADE: critically low, low, moderate, high. No effect: no statistical difference between traditional Chinese exercises and controls. Unclear effect: results showed mixed findings. Positive effect: effect estimates of traditional Chinese exercises are significantly positive. The bubble labels of T, B, W, L and Y represent Tai Chi, Baduanjin, Wuqinxi, Liuzijue and Yijinjing. | |
|  | **General summary** | **Effect** |
|  | **① Interventions:**  Liuzijue, Tai Chi, Baduanjin and Wuqinxi;  **② Diseases/symptoms:**  Chronic obstructive pulmonary disease (COPD);  **③ Effects and evidence quality:**  82.6% of SRs indicated clinical benefits of traditional Chinese exercises in COPD with critically low to low quality evidence. | **1. Positive effect:**  **1.1 Wuqinxi:**  **COPD:** Motor function: 6-minute walking test (6MWD); pulmonary function: forced expiratory volume in the first second (FEV1), forced vital capacity (FVC) and the ratio of forced expiratory volume in the first second to forced vital capacity (FEV1/FVC). |
|  |  | **2. Unclear effect:**  **Tai Chi, Baduanjin and Liuzijue on COPD**  **(**A total of eight SRs reported on Liuzijue, of which seven reported positive effects on COPD, but one reported beneficial effects on 6MWD and FEV1, and no statistically significant effects on FVC and FEV1/FVC of COPD.**)** |
| **3.6.5 Mental, behavioral, or neurodevelopmental disorders**  **(included 19 SRs)** | **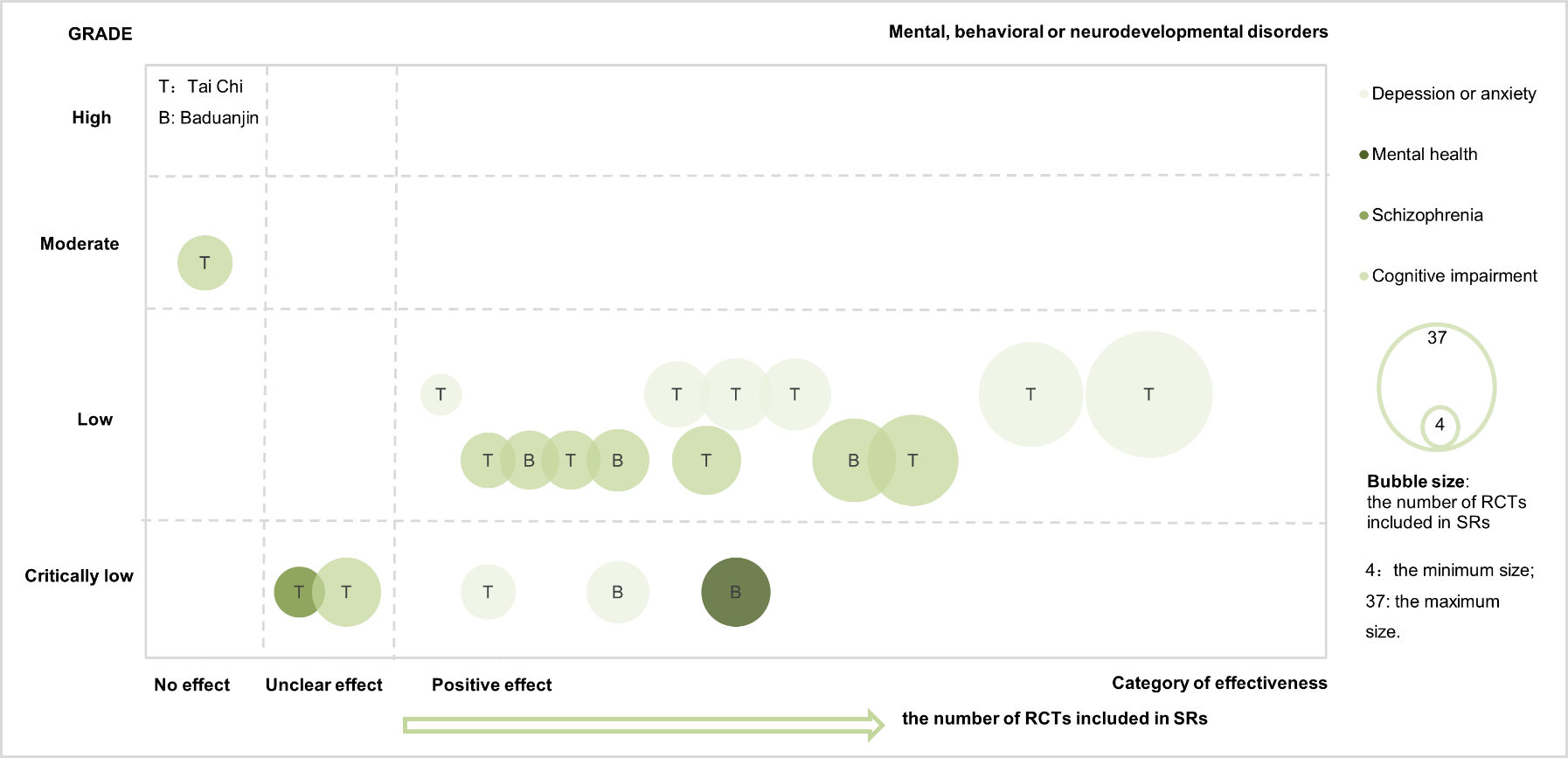**  **Supplementary Figure 8.** The evidence map of traditional Chinese exercises for mental, behavioral or neurodevelopmental disorders.  Note: The size of bubbles is determined by the number of RCTs included in SRs. The color of bubbles is represented different diseases and conditions. The level of GRADE: critically low, low, moderate, high. No effect: no statistical difference between traditional Chinese exercises and controls. Unclear effect: results showed mixed findings. Positive effect: effect estimates of traditional Chinese exercises are significantly positive. The bubble labels of T, B, W, L and Y represent Tai Chi, Baduanjin, Wuqinxi, Liuzijue and Yijinjing. | |
|  | **General summary** | **Effect** |
|  | **① Interventions:**  Tai Chi and Baduanjin  **② Diseases/symptoms:**  Cognitive impairment, depression or anxiety, schizophrenia and mental health  **③ Effects and evidence quality:**  84.2% of the SRs demonstrated clinical benefits of traditional Chinese exercises for these diseases, and the quality of evidence was critically low to moderate. | **1. Positive effect:**  **1.1 Tai Chi:**  **1.1.1 Anxiety, depression; mental health.**  **1.2 Baduanjin:**  **1.2.1 Anxiety, depression; mental health;**  **1.2.2 Cognitive impairment:** globle cognitive function. |
|  |  | **2. Unclear effect:**  **2.1 Tai Chi:**  **2.1.1 Cognitive impairment;**  **2.1.2 Schizophrenia** (Tai Chi was effective for schizophrenia on negative symptoms of the Positive and Negative Syndrome Scale but not on positive symptoms) |
| **3.6.6 Nervous system diseases**  **(included 17 SRs)** | **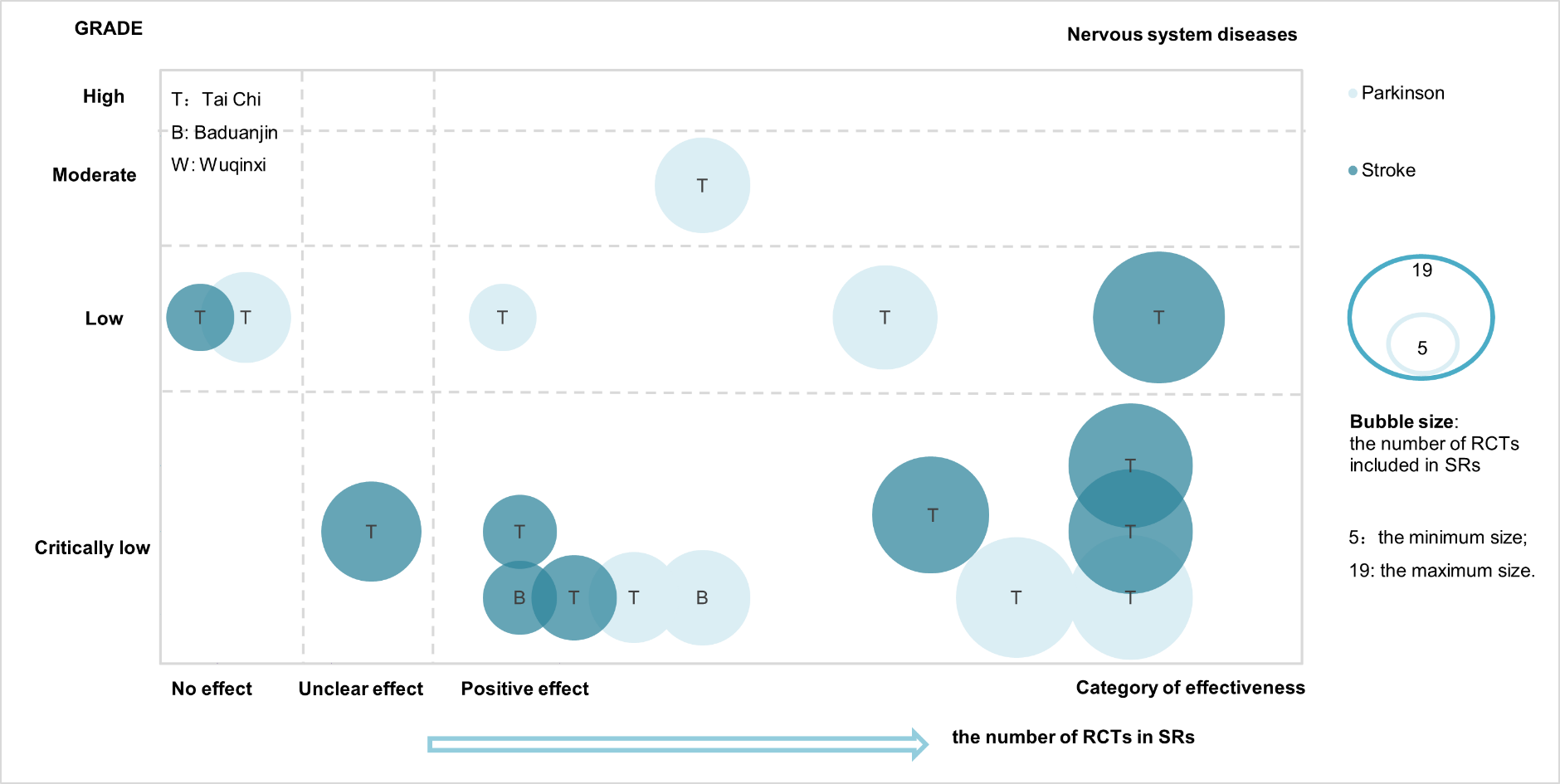**  **Supplementary Figure 9.** The evidence map of traditional Chinese exercises for nervous system diseases.  Note: The size of bubbles is determined by the number of RCTs included in SRs. The color of bubbles is represented different diseases and conditions. The level of GRADE: critically low, low, moderate, high. No effect: no statistical difference between traditional Chinese exercises and controls. Unclear effect: results showed mixed findings. Positive effect: effect estimates of traditional Chinese exercises are significantly positive. The bubble labels of T, B, W, L and Y represent Tai Chi, Baduanjin, Wuqinxi, Liuzijue and Yijinjing. | |
|  | **General summary** | **Effect** |
|  | **① Interventions:**  Tai Chi and Baduanjin;  **② Diseases/symptoms:**  Stroke, Parkinson’s disease;  **③ Effects and evidence quality:**  82.3% of the SRs indicated clinical benefits of traditional Chinese exercises in these diseases, and the quality of evidence was critically low to moderate. | **1. Positive effect:**  **1.1 Baduanjin:**  **1.1.1: Parkinson's disease:** Total UPDRS-III scores for daily living, motor function and motor complications;  **1.1.2: Stroke:** balance function. |
|  |  | **2. Unclear effect:**  **2.1 Tai Chi:**  **2.1.1 Parkinson's disease and stroke:** balance function |
| **3.6.7 Neoplasms**  **(included 9 SRs)** | **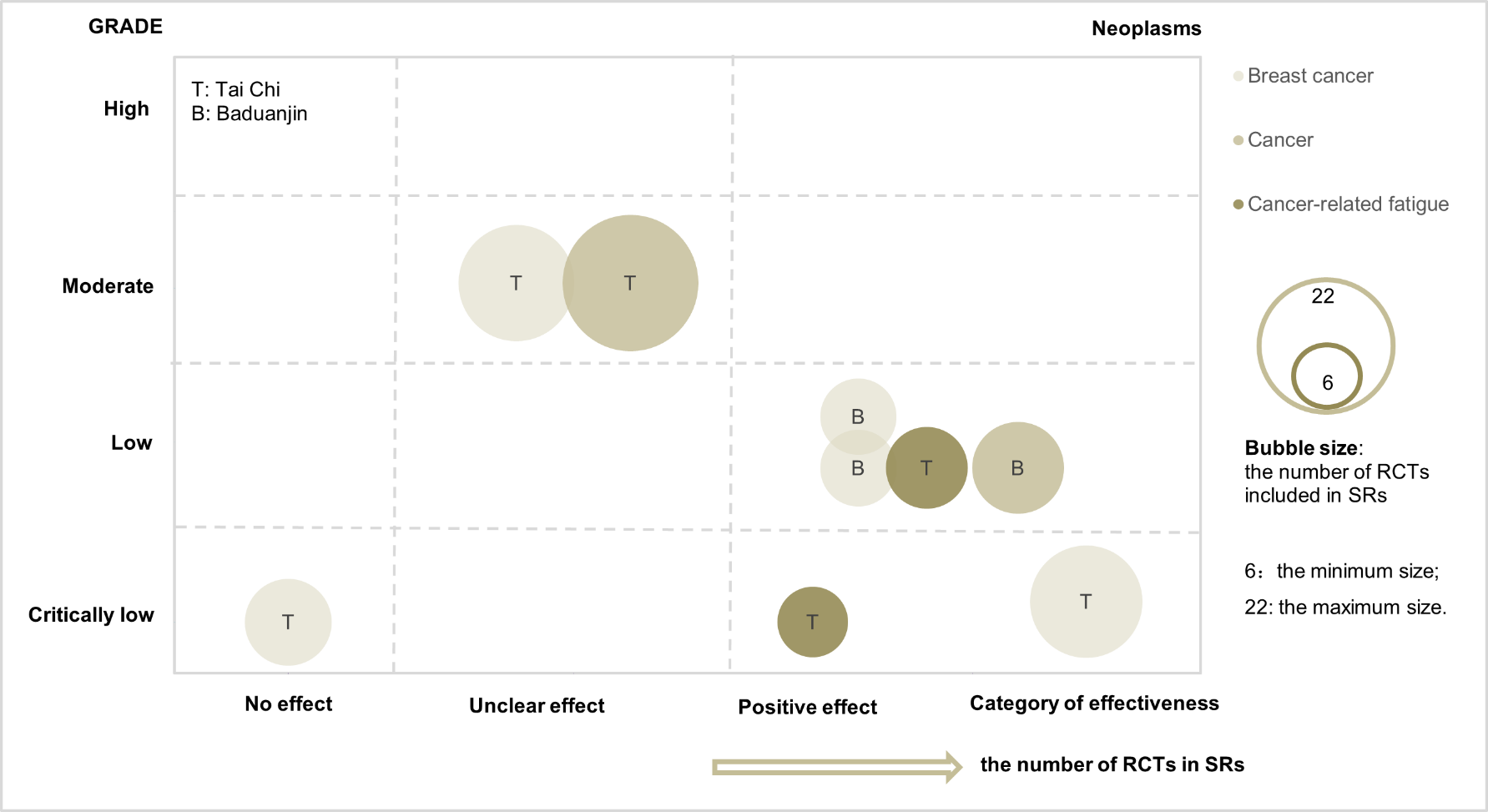**  **Supplementary Figure 10.** The evidence map of traditional Chinese exercises for neoplasms.  Note: The size of bubbles is determined by the number of RCTs included in SRs. The color of bubbles is represented different diseases and conditions. The level of GRADE: critically low, low, moderate, high. No effect: no statistical difference between traditional Chinese exercises and controls. Unclear effect: results showed mixed findings. Positive effect: effect estimates of traditional Chinese exercises are significantly positive. The bubble labels of T, B, W, L and Y represent Tai Chi, Baduanjin, Wuqinxi, Liuzijue and Yijinjing. | |
|  | **General summary** | **Effect** |
|  | **① Interventions:**  Tai Chi and Baduanjin;  **② Diseases/symptoms:**  Cancer and cancer-related fatigue;  **③ Effects and evidence quality:**  66.7% of SRs indicated clinical benefits of traditional Chinese exercises in these diseases with critically low to moderate quality evidence | **1. Positive effect:**  **1.1 Baduanjin:**  **1.1.1 Cancer:** the quality of life and fatigue. |
|  |  | **2. Unclear effect:**  **2.1 Tai Chi: cancer** |
| **3.6.8 Sleep-wake disorders**  **(included 8 SRs)** | **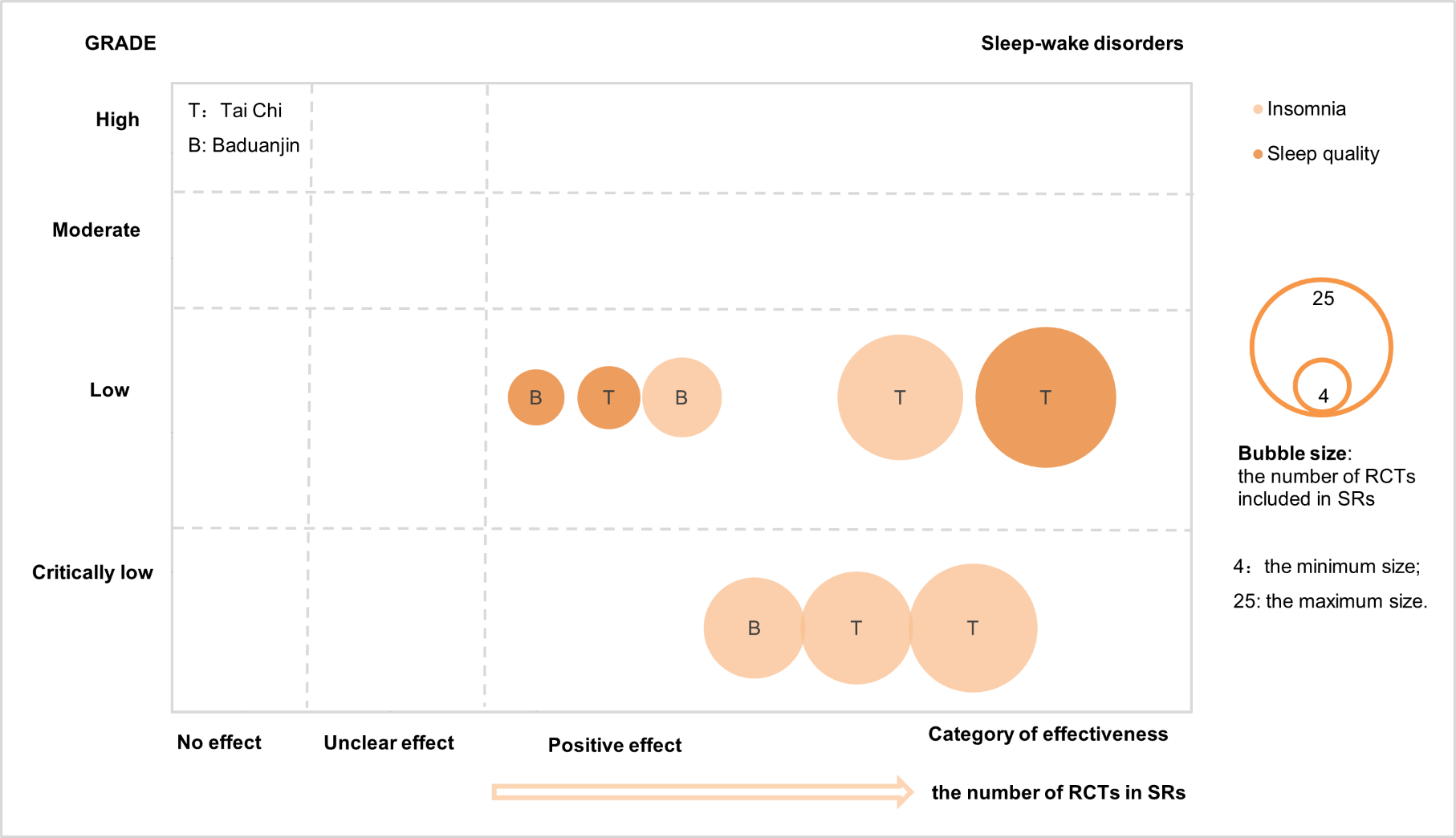**  **Supplementary Figure 11.** The evidence map of traditional Chinese exercises for sleep-wake disorders.  Note: The size of bubbles is determined by the number of RCTs included in SRs. The color of bubbles is represented different diseases and conditions. The level of GRADE: critically low, low, moderate, high. No effect: no statistical difference between traditional Chinese exercises and controls. Unclear effect: results showed mixed findings. Positive effect: effect estimates of traditional Chinese exercises are significantly positive. The bubble labels of T, B, W, L and Y represent Tai Chi, Baduanjin, Wuqinxi, Liuzijue and Yijinjing. | |
|  | **General summary** | **Effect** |
|  | **① Interventions:**  Tai Chi and Baduanjin  **② Diseases/symptoms:** sleep quality; insomnia  **③ Effects and evidence quality:** all the interventions showed positive effects with critically low to low quality | **1. Positive effect:**  **1.1 Tai Chi and Baduanjin: sleep quality; insomnia** |
| **3.6.9 Others**  **(included 21 SRs)** | **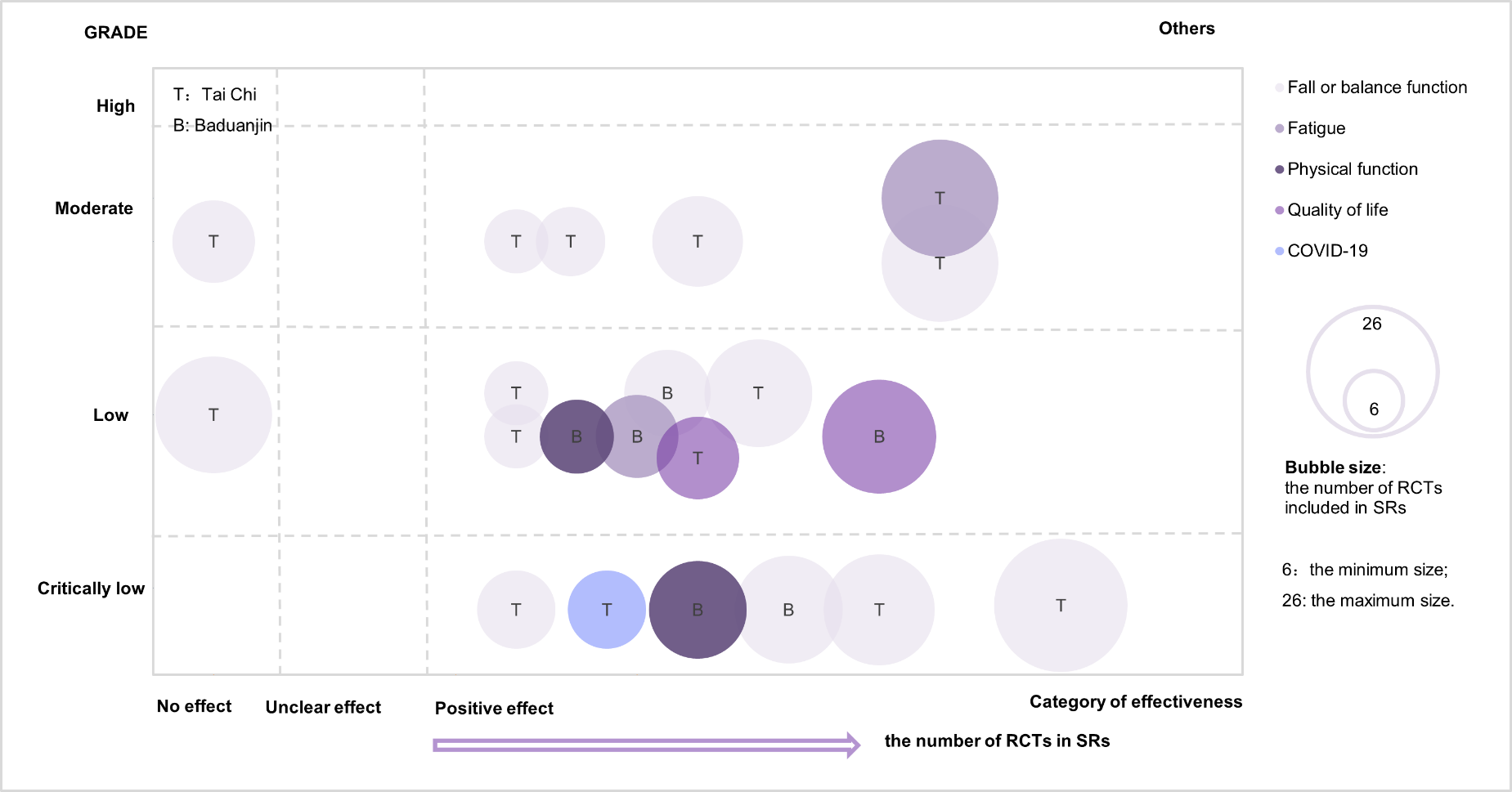**  **Supplementary Figure 12.** The evidence map of traditional Chinese exercises for others.  Note: The size of bubbles is determined by the number of RCTs included in SRs. The color of bubbles is represented different diseases and conditions. The level of GRADE: critically low, low, moderate, high. No effect: no statistical difference between traditional Chinese exercises and controls. Unclear effect: results showed mixed findings. Positive effect: effect estimates of traditional Chinese exercises are significantly positive. The bubble labels of T, B, W, L and Y represent Tai Chi, Baduanjin, Wuqinxi, Liuzijue and Yijinjing. | |
|  | **General summary** | **Effect** |
|  | **① Interventions:**  Tai Chi and Baduanjin  **② Diseases/symptoms:**  Balance function, fatigue, physical function, quality of life and COVID-19.  **③ Effects and evidence quality:**  90.5% of the SRs demonstrated clinical benefits of traditional Chinese exercises for these diseases/symptoms, and the quality of evidence was critically low to moderate. | **1. Positive effect:**  **1.1 Tai Chi:**  **1.1.1 Symptoms of fatigue and quality of life;**  **1.1.2 COVID-19:** outcomes of inflammation: C-reactive protein and TNF-alpha.  **1.2 Baduanjin:**  **1.2.1 Balance function, physical function and quality of life** |
|  |  | **2. Unclear effect:**  **2.1 Tai Chi:** balance function and fall risk |
