# Supplementary material for: Evidence map of traditional Chinese exercises
Source: Front Public Health. 2024 Sep 18;12:1347201. doi: 10.3389/fpubh.2024.1347201 (PMC11445016; doi:10.3389/fpubh.2024.1347201)
Supplement: Supplementary file 10 [file Table_6.docx]

**Supplementary Table 6: Characteristics and mechanisms of traditional Chinese exercises**

| **Interventions** | **Characteristic** | **Diseases/Conditions** | **Mechanism** |
| --- | --- | --- | --- |
| **Tai Chi**(1) | ① Moderate-intensity aerobic exercise;  ② Comprising a series of slow, gentle and multi-directional weight-bearing movements. | Musculoskeletal system and connective tissue disorders;  Parkinson’s disease;  Fall risk and balance function;  Stroke | 1. Overall regulation(2)   - Enhance the motor coordination and movement capacity - Improve gait and postural stability - Promote muscle strength and trunk flexibility   2. Improve lower and upper limbs movement(3, 4)  2.1 Lower limbs:   - Improve balance function - Improve the muscle strength of the hip joint, knee joint, and ankle joint   2.2 Upper limbs:   - Exercise the joints and ligaments coherently   3. Improve nerve system(5, 6)   - Improve blood circulation, reshape and compensate nerve function to form new neural pathways - Stimulate the motor sensory area of the brain |
|  |  | Hypertension | 1. Reduce blood pressure(7, 8)   - Increase the excitability of parasympathetic nerves - Improve vascular compliance and cause vasodilation - Modulate pressure receptors in the carotid sinus and aortic arch - Dilate coronary arteries   2. Reduce the risk of hypertension(9)   - Improve the nitric oxide bioavailability - Modulate structural proteins - Reduce oxidative stress and inflammation - Prevent elastic artery stiffness and endothelial dysfunction |
|  |  | Depression;  Anxiety;  Chronic pain;  Sleep disorders | 1. Improve cognitive function and exert anti-depressant effects(10-12)   - Lengthen telomeres, increase telomerase activity and prefrontal cortex gray matter volume as well as hippocampal volume - Reduce neuroinflammation   2. Improve negative psychological symptoms(13)   - Enhance autonomic regulation and parasympathetic nerves, - Promote the body to secrete norepinephrine and dopamine substances |
| **Baduanjin(14)** | ① A low-intensity aerobic exercise;  ② Consists of eight separate different movements;  ③ Has the effects of dredging meridians and collaterals, facilitating qi and blood circulation, regulating the internal organs and keeping fit through musculoskeletal movements, meditation minds, and breathing techniques. | Diabetes;  Hyperlipidemia | 1. Improve glucose metabolism(15, 16)   - Increase the ability of insulin to stimulate glucose transporter type 4 (GLUT4) in muscle cells - Improve muscle absorption, transportation and utilization of lipids and glucose. - Regulate the expression of mRNA, IncRNA, and circRNA(17) - Improve lipoprotein protease activity and the ability of the skeletal muscle to utilize fatty acid supply   2. Improve lipid metabolism(18, 19)   - Improve lipoprotein protease activity and the ability of the skeletal muscle to utilize fatty acid supply |
|  |  | Coronary heart diseases | 1. Reduce heart burden(20)   - Improve the ability to transport and utilize oxygen in blood circulation   2. Improve the hypoxia state(21-23)   - Exert cardiorespiratory function by regulating parasympathetic activity and heart rate variability - Increase exercise endurance by breathing   3. Alleviate pathological LV remodeling(20, 24, 25)   - Alleviate oxidative stress injury - Protect endothelial function and blood vessel elasticity - Mitigate ischemia/reperfusion-induced heart damage |
| **Liuzijue(26-28)** | ① Mainly focuses on breathing, supplemented by body movements.  ② Not only uses deep, slow, vocal and reverse abdominal breathing to exercise the function of the respiratory muscles, but also uses physical movements to exercise the function of skeletal muscles. | Chronic obstructive pulmonary disease | 1. Increase pulmonary ventilation function(29, 30)   - Mobilize respiratory muscles such as the intercostal muscle, rectus abdominis muscle, and pectoralis major muscle - Increase the movement range of the diaphragm rise and fall and enhance the strength of respiratory muscle - Extend expiratory time, deepen respiratory depth, improve the airway pressure   2. Integral regulation(30, 31)   - Enhance the flexibility, coordination, and control capacity of neuromuscular - Improve the T cell immune function |
| **Wuqinxi(32)** | ① An all-round exercise of human body based on five typical movements of tigers, deers, bears, apes, and birds corresponding to the human body's five internal organs.  ② The movements are comprehensive, involving the major muscle groups of the whole body. | Osteoarthritis;  Respiratory diseases | 1. Improving pulmonary ventilation(33)   - Upper-limb stretching or thoracic expansion coordinated with deep breathing - Modulate the diaphragmatic and intercostal muscles - Maintaining the continuity and patency of breathing   2. Improve musculoskeletal system(34)   - Improve the blood circulation of the soft tissue of the spine and other joints - Improve the excitability of the action neurons - Strengthen the muscles - Maintain the normal structure of bones and joints |
| **Yijinjing(35, 36)** | ① A moderate-intensity exercise emphasizing the combination of symmetrical physical postures, consciousness, and breathing. | Musculoskeletal system | 1. Improve musculoskeletal system(37, 38)   - Improve the strength, flexibility and balance of the muscles and ligaments - Improve the stability of the body |

**References**

1. Law NY, Li JX, Zhu Q, Nantel J. Effects of a biomechanical-based Tai Chi program on gait and posture in people with Parkinson's disease: study protocol for a randomized controlled trial. Trials. 2023;24(1):241.

2. Lim CL, Keong NLS, Yap MMC, Tan AWK, Tan CH, Lim WS. The effects of community-based exercise modalities and volume on musculoskeletal health and functions in elderly people. Front Physiol. 2023;14:1227502.

3. Kim H, Kim YL, Lee SM. Effects of therapeutic Tai Chi on balance, gait, and quality of life in chronic stroke patients. Int J Rehabil Res. 2015;38(2):156-61.

4. Stephenson JL, Lamontagne A, De Serres SJ. The coordination of upper and lower limb movements during gait in healthy and stroke individuals. Gait Posture. 2009;29(1):11-6.

5. He J, Li C, Lin J, Shu B, Ye B, Wang J, et al. Proprioceptive Training with Visual Feedback Improves Upper Limb Function in Stroke Patients: A Pilot Study. Neural Plast. 2022;2022:1588090.

6. Chen Y, Wan A, Mao M, Sun W, Song Q, Mao D. Tai Chi practice enables prefrontal cortex bilateral activation and gait performance prioritization during dual-task negotiating obstacle in older adults. Front Aging Neurosci. 2022;14:1000427.

7. Hartley L, Flowers N, Lee MS, Ernst E, Rees K. Tai chi for primary prevention of cardiovascular disease. Cochrane Database Syst Rev. 2014(4):Cd010366.

8. Pan X, Tian L, Yang F, Sun J, Li X, An N, et al. Tai Chi as a Therapy of Traditional Chinese Medicine on Reducing Blood Pressure: A Systematic Review of Randomized Controlled Trials. Evid Based Complement Alternat Med. 2021;2021:4094325.

9. Korsager Larsen M, Matchkov VV. Hypertension and physical exercise: The role of oxidative stress. Medicina (Kaunas). 2016;52(1):19-27.

10. Wetherell JL, Hershey T, Hickman S, Tate SR, Dixon D, Bower ES, et al. Mindfulness-Based Stress Reduction for Older Adults With Stress Disorders and Neurocognitive Difficulties: A Randomized Controlled Trial. J Clin Psychiatry. 2017;78(7):e734-e43.

11. Wu C, Feng Y. Exploring the potential of mindfulness-based therapy in the prevention and treatment of neurodegenerative diseases based on molecular mechanism studies. Front Neurosci. 2023;17:1097067.

12. Wolkowitz OM, Mellon SH, Lindqvist D, Epel ES, Blackburn EH, Lin J, et al. PBMC telomerase activity, but not leukocyte telomere length, correlates with hippocampal volume in major depression. Psychiatry Res. 2015;232(1):58-64.

13. Du Z, Zhang X, Qin H, Wang R, Bai Y, Yao X. META analysis on the effect of taijiquan on improving negative psychological symptoms of college students and the optimal dose. Front Public Health. 2022;10:1032266.

14. Qian H, Xu W, Cui L, Wang R, Wang J, Tang M, et al. Efficacy of Bushen Huatan Decoction combined with Baduanjin in the treatment of polycystic ovary syndrome with insulin resistance (IR-PCOS), kidney deficiency and phlegm dampness: study protocol for a randomized controlled trial. Trials. 2021;22(1):781.

15. Kong L, Ren J, Fang S, He T, Zhou X, Fang M. Effects of traditional Chinese mind-body exercise-Baduanjin for type 2 diabetes on psychological well-being: A systematic review and meta-analysis. Front Public Health. 2022;10:923411.

16. Ma X, Li M, Liu L, Lei F, Wang L, Xiao W, et al. A randomized controlled trial of Baduanjin exercise to reduce the risk of atherosclerotic cardiovascular disease in patients with prediabetes. Sci Rep. 2022;12(1):19338.

17. An T, He ZC, Zhang XQ, Li J, Chen AL, Tan F, et al. Baduanjin exerts anti-diabetic and anti-depression effects by regulating the expression of mRNA, lncRNA, and circRNA. Chin Med. 2019;14:3.

18. Ma Q, Li H, Gao Y, Zou Y. Effects of Baduanjin on glucose and lipid metabolism in diabetic patients: A protocol for systematic review and meta-analysis. Medicine (Baltimore). 2021;100(4):e23532.

19. Muscella A, Stefàno E, Marsigliante S. The effects of exercise training on lipid metabolism and coronary heart disease. Am J Physiol Heart Circ Physiol. 2020;319(1):H76-h88.

20. Mao S, Zhang X, Shao B, Hu X, Hu Y, Li W, et al. Baduanjin Exercise Prevents post-Myocardial Infarction Left Ventricular Remodeling (BE-PREMIER trial): Design and Rationale of a Pragmatic Randomized Controlled Trial. Cardiovasc Drugs Ther. 2016;30(3):315-22.

21. Chen DM, Yu WC, Hung HF, Tsai JC, Wu HY, Chiou AF. The effects of Baduanjin exercise on fatigue and quality of life in patients with heart failure: A randomized controlled trial. Eur J Cardiovasc Nurs. 2018;17(5):456-66.

22. Yang WY, Xu Y, Ye L, Rong LJ, Feng J, Huang BL, et al. Effects of Baduanjin exercise on quality-of-life and exercise capacity in patients with heart failure: A systematic review and meta-analysis. Complement Ther Clin Pract. 2023;50:101675.

23. Xu J, Zhang Z, Liu J, Li Y, Wan J, Feng R, et al. Effect of traditional Asian exercise on patients with chronic heart failure: a protocol for network meta-analysis of randomised controlled trials. BMJ Open. 2021;11(8):e048891.

24. Chen M, Ou L, Chen Y, Men L, Zhong X, Yang S, et al. Effectiveness and safety of Baduanjin exercise (BDJE) on heart failure with preserved left ventricular ejection fraction (HFpEF): A protocol for systematic review and meta-analysis. Medicine (Baltimore). 2020;99(46):e22994.

25. Mao S, Zhang X, Chen M, Wang C, Chen Q, Guo L, et al. Beneficial Effects of Baduanjin Exercise on Left Ventricular Remodelling in Patients after Acute Myocardial Infarction: an Exploratory Clinical Trial and Proteomic Analysis. Cardiovasc Drugs Ther. 2021;35(1):21-32.

26. Hu J, Gao R, Wang Y, Li Y, Wang Y, Wang Z, et al. Effect of Liuzijue on pulmonary rehabilitation in patients with chronic obstructive pulmonary disease: study protocol for a multicenter, non-randomized, prospective study. BMC Complement Med Ther. 2022;22(1):296.

27. Li L, Huang H, Song J, Yu Y, Jia Y, Wang Y, et al. Network Meta-Analysis of the Effects of Different Types of Traditional Chinese Exercises on Pulmonary Function, Endurance Capacity and Quality of Life in Patients With COPD. Front Med (Lausanne). 2022;9:806025.

28. Xu S, Zhang D, He Q, Ma C, Ye S, Ge L, et al. Efficacy of Liuzijue Qigong in patients with chronic obstructive pulmonary disease: A systematic review and meta-analysis. Complement Ther Med. 2022;65:102809.

29. Yi J, Wang F, Yue R, Lin Q, Ding R, Xie X, et al. Effect of sitting and lying Liuzijue for lung rehabilitation in acute exacerbation of chronic obstructive pulmonary disease patients with non-invasive ventilation: Study protocol for a randomized controlled trial. Medicine (Baltimore). 2020;99(38):e22111.

30. Gao P, Tang F, Liu W, He K, Mo Y. Effect of liuzijue qigong on patients with stable chronic obstructive pulmonary disease: A systematic review and meta-analysis. Medicine (Baltimore). 2021;100(41):e27344.

31. Li P, Liu J, Lu Y, Liu X, Wang Z, Wu W. Effects of long-term home-based Liuzijue exercise combined with clinical guidance in elderly patients with chronic obstructive pulmonary disease. Clin Interv Aging. 2018;13:1391-9.

32. Chang XQ, Chen XP, Shen YX, Wang K, Huang SJ, Qi Y, et al. The deer play in Wuqinxi and four-point hand-knee kneeling positions for training core muscle function and spinal mobility. Front Bioeng Biotechnol. 2022;10:965295.

33. Yu F, Xin M, Liu N, Huang N, Lu J. The Qigong Wuqinxi for chronic obstructive pulmonary disease: Protocol for a systematic review and meta-analysis. Medicine (Baltimore). 2019;98(30):e16633.

34. Xiao Z, Li G. The effect of Wuqinxi exercises on the balance function and subjective quality of life in elderly, female knee osteoarthritis patients. Am J Transl Res. 2021;13(6):6710-6.

35. Gao H, Luo C, Tu SJ, Lu RP, Jiang LN, Qiao HJ, et al. The Effect of Yijinjing on the Cognitive Function of Patients With Chronic Schizophrenia. Front Psychiatry. 2021;12:739364.

36. Chen Y, Ma Y, Zhang Z, Zhang Y, Jia J. The efficacy and safety of Yijinjing exercise in the adjuvant treatment of ankylosing spondylitis: A protocol of randomized controlled trial. Medicine (Baltimore). 2021;100(38):e27109.

37. Zhuang Q, Feng H, Jing F, Rong J, Lv Y, Jing F, et al. Effect of Yijinjing exercise on cervical spondylosis: A protocol for systematic review. Medicine (Baltimore). 2020;99(27):e20764.

38. Huang Y, Han J, Gu Q, Cai Y, Li J, Wang S, et al. Effect of Yijinjing combined with elastic band exercise on muscle mass and function in middle-aged and elderly patients with prediabetes: A randomized controlled trial. Front Med (Lausanne). 2022;9:990100.
